# Summary of the best evidence for non-surgical intervention in periodontitis among patients with type 2 diabetes mellitus

**DOI:** 10.3389/fpubh.2026.1792923

**Published:** 2026-04-20

**Authors:** Huiying Sha, Huiling Xu, Houjuan Zu, Shan Fan, Xiangjie Shen, Jingjing Wang, Wei Yin, Hongbing Bu, Qiaoyan Liu

**Affiliations:** 1 Department of Endocrinology, Affiliated Hospital of Jiangsu University, Zhenjiang, Jiangsu, China; 2 School of Medicine, Jiangsu University, Zhenjiang, Jiangsu, China

**Keywords:** type 2 diabetes mellitus, periodontitis, non-surgical intervention, complications, evidence summary

## Abstract

**Objective:**

To summarize the best evidence for non-surgical intervention in periodontitis among patients with type 2 diabetes mellitus, and to provide a basis for medical and nursing staff to carry out periodontitis management in diabetic patients.

**Methods:**

Based on the 5S model, a literature search was conducted in UpToDate, BMJ Best Practice, the Joanna Briggs Institute (JBI) Evidence-Based Healthcare Center Database, the website of the National Institute for Health and Care Excellence (NICE), guideline networks, and Chinese and international databases to collect clinical decisions, guidelines, evidence summaries, expert consensuses, systematic reviews, and Meta-analyses related to non-surgical intervention for periodontitis in patients with type 2 diabetes mellitus. The search period spanned from the establishment of each database/website up to March 9, 2026. Quality assessment of the retrieved literature was performed, followed by evidence extraction and synthesis of the finally included studies.

**Results:**

A total of 17 studies were included, consisting of 3 clinical decision documents, 5 guidelines, 2 evidence summaries, 3 expert consensuses, and 4 systematic reviews. Finally, 27 pieces of best evidence were summarized from 7 aspects, including periodontitis assessment, risk factor control, oral hygiene guidance, dietary management, mechanical intervention, adjuvant measures, and follow-up management.

**Conclusion:**

This study summarizes the best evidence for non-surgical intervention in periodontitis among patients with type 2 diabetes mellitus, providing an evidence-based basis for clinical practice. Clinical medical and nursing professionals may reasonably apply this evidence according to clinical scenarios and patients’ preferences to prevent the occurrence of periodontitis in diabetic patients and restore a healthy periodontal environment.

## 1 Introduction

Periodontitis is a chronic inflammatory disease characterized by the destruction of periodontal supporting connective tissues, caused by the dysbiosis of oral microbiota, and it is a common oral complication in patients with diabetes mellitus (1). A survey conducted in China showed that the prevalence rate of periodontitis among patients with type 2 diabetes mellitus is as high as 73.14% (2). A bidirectional pathogenic association exists between periodontitis and type 2 diabetes mellitus. A hyperglycemic environment exacerbates the inflammatory response in periodontal tissues, leading to tooth mobility, tooth loss, and even speech impairment. In turn, periodontitis induces a systemic inflammatory response, which maintains blood glucose at a high level and increases the risk of cardiovascular lesions—thus forming a vicious cycle (3, 4). For patients with type 2 diabetes mellitus, adequate blood glucose control and professional oral hygiene guidance can prevent the development of periodontitis. For patients with mild to moderate periodontitis, non-surgical intervention measures—such as enhanced oral hygiene guidance combined with the use of cleaning tools—can achieve the effect of inflammatory control. For patients with severe periodontitis, non-surgical intervention is mostly used as the basis; if the control effect is unsatisfactory, surgical intervention should be considered to alleviate the periodontal inflammatory state (5). Relevant studies have shown that patients with diabetes mellitus complicated by periodontitis should adhere to the treatment principle of multiple, short-duration, and non-surgical periodontal interventions, which can alleviate periodontal inflammation while effectively improving blood glucose levels (6). Currently, evidence regarding non-surgical interventions for periodontitis in patients with type 2 diabetes mellitus is relatively scattered internationally; Domestic literature mainly focuses on the analysis of influencing factors and research on the pathological mechanisms of periodontitis (7, 8). At present, clinical medical staff lack standardized assessment and systematic intervention procedures for this specific population. This study aims to systematically search and summarize the best evidence on non-surgical intervention for periodontitis in patients with type 2 diabetes mellitus at home and abroad, so as to provide an evidence-based basis for medical staff to implement clinical practice.

## 2 Materials and methods

### 2.1 Establishment of the research question

The evidence-based question was constructed according to the PIPOST model (9), with the following components: P (Population)—Target Population: Patients with type 2 diabetes mellitus; patients with type 2 diabetes mellitus complicated by periodontitis. I (Intervention)—Intervention Methods: Assessment, risk factor control, oral hygiene guidance, follow-up, and other related measures. P (Professional)—Personnel for Evidence Application: Administrators, endocrinologists, stomatologists, and nurses. O (Outcome)—Outcome Indicators: Periodontal-related indices (e.g., plaque index, bleeding index, probing depth); metabolic-related indicators (e.g., blood glucose, glycated hemoglobin); oral health-related quality of life. S (Setting)—Settings for Evidence Application: Endocrinology department, stomatology department, and home. T (Type of Evidence)—Type of Evidence: Clinical decision documents, guidelines, evidence summaries, expert consensuses, systematic reviews, and Meta-analyses.

### 2.2 Search strategy

Based on the “5S” search model (10), a hierarchical search was conducted across the following databases and websites: UpToDate, BMJ Best Practice, Joanna Briggs Institute (JBI) Evidence-Based Healthcare Center Database, National Institute for Health and Care Excellence (NICE) Website, Registered Nurses’ Association of Ontario (RNAO) Website, Guidelines International Network (GIN), Canadian Collaboration for Healthcare Improvement (CCHI), American Dental Association (ADA), World Dental Federation (FDI), International Association for Dental Research (IADR), Chinese Stomatological Association (CSA), Chinese Medical Knowledge Database, Yimaitong Guidelines Network, Cochrane Library, CINAHL, PubMed, Embase, China National Knowledge Infrastructure (CNKI), and Wanfang Database. The search terms were: “periodont* or periodontitis” and “type 2 diabetes mellitus” and “oral health or oral hygiene or oral care or prevention or management or treatment or evaluat*or assess*or Oral Health” and “best practice or guideline or evidence summar*or consensus or expert opinion or systematic review or Meta analysis”. In this study, the literature retrieval was performed using a combination of subject headings and free-text terms. The search period was from the establishment of each database up to March 9, 2026. The summarized evidence was comprehensive, and no primary studies were retrieved. The detailed search strategy is provided in the Supplementary material 1.

### 2.3 Inclusion and exclusion criteria for literature

Inclusion Criteria: ① The study population consists of patients with type 2 diabetes mellitus aged ≥ 18 years. ② The study content involves research on non-surgical interventions for periodontitis in patients with type 2 diabetes mellitus. ③ The study types include clinical decision documents, guidelines, evidence summaries, expert consensuses, systematic reviews, and Meta-analyses. ④ The study languages are Chinese or English.

Exclusion Criteria: ① The study types are conference abstracts or studies for which the full text is unavailable. ② Duplicated published literature.

### 2.4 Literature quality evaluation

Clinical practice guidelines were evaluated using the Appraisal of Guidelines for Research & Evaluation II (AGREE II) (11). This evaluation tool comprises 6 domains, 23 items, and 2 overall assessment items. Each item is scored on a scale of 1 to 7, and the total score for each domain is calculated using a formula for standardized percentage, based on which the final rating is determined. Clinical decision documents and evidence summaries were evaluated using the Critical Appraisal for Summaries of Evidence (CASE) tool (12). This tool covers 4 aspects with a total of 10 items, and each item has 3 evaluation criteria: “Yes,” “No,” and “Partially Yes.” A study was excluded if more than 5 items were rated “No,” or if the proportion of items rated “Partially Yes” was ≥ 60%. Expert consensuses were evaluated using the validity assessment tool developed by the Joanna Briggs Institute (JBI) for Evidence-Based Healthcare (13), which includes 6 items. Each item uses the following assessment criteria: “Yes,” “No,” “Unclear,” and “Not Applicable.” Systematic reviews were evaluated using the systematic review assessment tool developed by the JBI for Evidence-Based Healthcare, covering 11 items (14). Each item also uses the assessment criteria of “Yes,” “No,” “Unclear,” and “Not Applicable.”Clinical guidelines were independently evaluated by 4 researchers, while other types of literature were evaluated by 2 researchers. All researchers had received systematic evidence-based training. When there were discrepancies in literature assessment opinions, the evidence-based team conducted in-group discussions, and 1 evidence-based expert was invited to make a judgment to decide whether to include the literature.

### 2.5 Evidence extraction, integration, and hierarchy grading

Two researchers independently extracted the evidence, and then collaborated with other members of the evidence-based team to translate, proofread, and integrate the relevant evidence. In this study, the 2014 version of the JBI Evidence Pre-grading System was used to classify the types of original literature of the included evidence, with the evidence grades ranging from Level 1 to Level 5. The recommendation strength of each piece of evidence was determined by integrating its validity, feasibility, appropriateness, and clinical evidence, together with the 2014 JBI Levels of Evidence. Grade A indicates a strong recommendation, while Grade B indicates a weak recommendation (15). Evidence content extracted from the literature included in the JBI Database directly adopts the original evidence levels. A meeting of experts was also convened, with 6 experts invited to integrate the evidence. The experts consisted of 2 endocrinologists, 2 provincial-level diabetes specialist nurses, 1 stomatologist, and 1 full-time stomatological nurse. The principles for summarizing evidence are as follows: ① Organize and merge complementary or similar evidence; ② When there is a conflict in the content of evidence, trace back to the original literature and follow the principles of prioritizing evidence-based evidence, high-quality evidence, and the latest published evidence for screening; ③ For evidence with consistent content, prioritize those expressed concisely and professionally.

## 3 Result

### 3.1 Literature screening process and results

**Figure 1:**
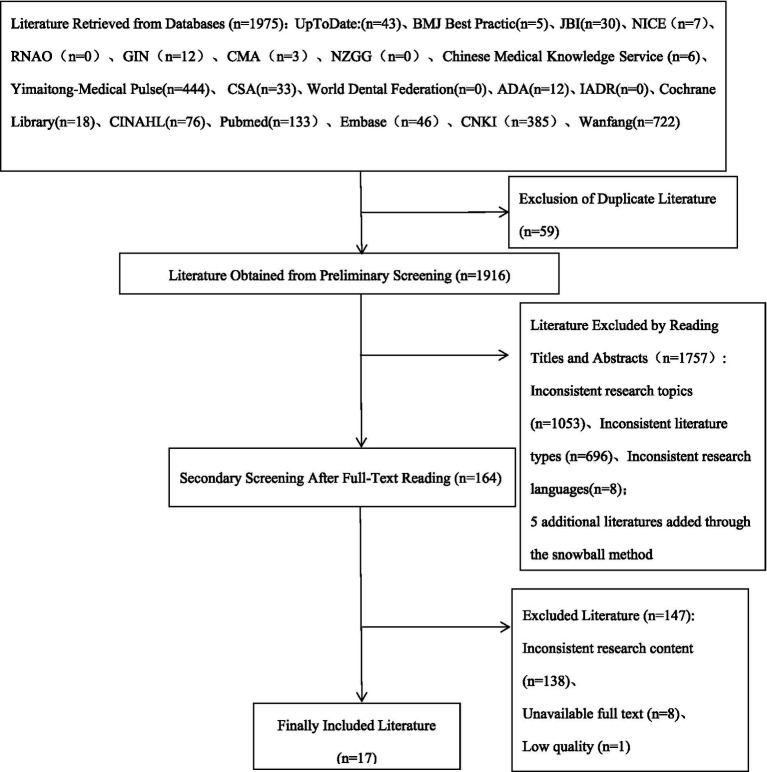
Flowchart of Literature Screening.

A total of 1975 articles were initially retrieved, and 17 articles were finally included. Among these included articles, there were 3 clinical decision-making documents, 5 guidelines, 2 evidence summaries, 3 expert consensuses, and 4 systematic reviews. The literature screening process is shown in Figure 1, and the general characteristics of the included articles are presented in Table 1.

**Table 1:** Basic characteristics of included literature.

Included literature	Literature reference	Year of publication (year)	Type of literature	The literature theme
Wexler (16)	UpToDate	2025	Clinical decision	Overview of general medical care in nonpregnant adults with diabetes mellitus
Chow (17)	UpToDate	2024	Clinical decision	Complications, diagnosis, and treatment of odontogenic infections
Wilder et al. (18)	UpToDate	2023	Clinical decision	Overview of gingivitis and periodontitis in adults
NICE (19)	NICE	2022	Guideline	Type 2 diabetes in adults: management
Health Improvement and Disparities (20)	NICE	2021	Guideline	Delivering better oral health: an evidence-based toolkit for prevention
Sanz et al. (21)	Pubmed	2020	Guideline	Treatment of stage I-III periodontitis-The EFP S3 level clinical practice guideline
NICE (22)	NICE	2018	Guideline	Oral health promotion: general dental practice
GIN (23)	GIN	2012	Guideline	Management of Chronic Periodontitis
Omid (24)	JBI	2023	Evidence Summary	Periodontal Disease (Prevention and Treatment): Oral Hygiene Practices
Fong (25)	JBI	2022	Evidence Summary	Periodontitis: Treatment
Adda et al. (26)	CINAHL	2021	Expert consensus	Consensus report of the joint workshop of the Italian Society of Diabetology, Italian Society of Periodontology and Implantology, Italian Association of Clinical Diabetologists (SID-SIdP-AMD)
Sanz et al. (27)	CINAHL	2018	Expert consensus	Scientific evidence on the links between periodontal diseases and diabetes: Consensus report and guidelines of the joint workshop on periodontal diseases and diabetes by the International Diabetes Federation and the European Federation of Periodontology
Society of Periodontology, Chinese Stomatological Association (28)	Chinese Medical Knowledge Service	2017	Expert consensus	Consensus of Chinese experts on diagnosis of severe periodontitis and treatment principles of periodontitis in special population
Corbella et al. (29)	CINAHL	2024	Systematic review	Efficacy of different protocols of non-surgical periodontal therapy in patients with type 2 diabetes: A systematic review and meta-analysis
Silva-Junior et al. (30)	Embase	2023	Systematic review	The effect of antimicrobial photodynamic therapy adjunct to non-surgical periodontal therapy on the treatment of periodontitis in individuals with type 2 diabetes mellitus: A systematic review and meta-analysis
Oliveira VB et al. (31)	Pubmed	2023	Systematic review	Effect of subgingival periodontal therapy on glycaemic control in type 2 diabetes patients: Meta-analysis and meta-regression of 6-month follow-up randomized clinical trials
Elisa Grillo. Araújo et al. (32)	Pubmed	2022	Systematic review	Efficacy of Antioxidant Supplementation to Non-Surgical Periodontal Therapy on Metabolic Control in Type 2 Diabetes Patients: A Network Meta-Analysis

### 3.2 Results of literature quality assessment

#### 3.2.1 Results of guideline quality assessment

A total of 5 guidelines were included (19–22, 24), and the results of quality assessment are presented in Table 2. The intraclass correlation coefficients (ICCs) were 0.886, 0.889, 0.892, 0.869, and 0.890 respectively, with *p* < 0.001, indicating good inter-rater consistency.

**Table 2:** Results of guideline assessment.

Guideline	Standardized scores in various domains (%)						≥60%	≥30%	Quality evaluation
	Scope and purpose	Stakeholder involvement	Rigour of development	Clarity of presentation	Applicability	Editorial independence			
NICE (19)	70.84%	77.78%	61.97%	58.3%	57.20%	93.75%	4	6	B
NICE (22)	79.10%	73.61%	61.46%	54.17%	60.42%	37.50%	4	6	B
Health Improvement and Disparities (20)	88.89%	77.78%	61.46%	80.56%	54.17%	81.25%	5	6	B
GIN (23)	83.3%	72.22%	47.40%	75%	37.50%	87.5%	4	6	B
Sanz M et al. (21)	81.95%	63.89%	65.10%	81.94%	45.83%	81.25%	5	6	B

#### 3.2.2 Results of quality assessment for clinical decision-making and evidence summaries

A total of 3 clinical decision-making documents (16–18) and 2 evidence summaries (24–25) were included, and the results of quality assessment are presented in Table 3.

**Table 3:** Results of clinical decision-making and evidence summary quality evaluation.

Items	UpToDate (16)	UpToDate (17)	UpToDate (18)	JBI (24)	JBI (25)
1. Is the summary specific in scope and application?	Yes	Yes	Yes	Yes	Yes
2. Is the authorship of the summary transparent?	Yes	Yes	Yes	Yes	Yes
3. Are the reviewer(s)/editor(s) of the summary transparent?	Yes	Yes	Yes	No	No
4. Are the search methods transparent and comprehensive?	No	No	No	No	No
5. Is the evidence graded and is the system transparent and translatable?	No	No	No	Yes	Yes
6. Are the recommendations clear?	Yes	Yes	Yes	Yes	Yes
7. Are the recommendations appropriately cited?	Yes	Yes	Yes	Yes	Yes
8. Are the recommendations current?	Yes	Yes	Yes	Yes	Yes
9. Is the summary free of possible bias?	Yes	Yes	Yes	Yes	Yes
10. Can this summary be applied to your patient(s)?	Yes	Yes	Yes	Yes	Yes

#### 3.2.3 Results of quality assessment for expert consensuses

A total of 3 expert consensuses (26–28) were included, and the evaluation result for all items was “Yes.”

#### 3.2.4 Results of quality assessment for systematic reviews

A total of 4 systematic reviews were included. For the studies by Elisa Grillo Araújo et al. (32) and Silva-Junior et al. (30), the evaluation result for Item 9 (“Whether potential publication bias was assessed”) was “No,” while the results for all other items were “Yes.” The remaining 2 articles received a “Yes” for all evaluation items. After discussion by the evidence-based team, these systematic reviews were determined to be of high quality and thus included in the study.

### 3.3 Evidence summary

Summary of the best evidence for non-surgical intervention in periodontitis among patients with type 2 diabetes mellitus (Table 4).

**Table 4:** Summary of the best evidence for non-surgical intervention in periodontitis among patients with type 2 diabetes mellitus.

Category	Content of evidence	Recommendation level	Grade
Assessment	1. Medical History Assessment: Assessment of diabetes history, including the duration of diabetes, the presence of existing diabetic complications, and diabetes treatment regimens; as well as histories of other diseases such as cancer and acquired immunodeficiency syndrome (AIDS); and the family history of periodontitis (25, 27).	5b	A
	2. Risk Factor Assessment: Evaluate the presence of plaque accumulation, smoking history, alcohol consumption history, and medication history (including antidepressants, calcium channel blockers, anticoagulants, antihypertensive drugs, statins, beta-blockers, and anticholinergic drugs) (20, 25–27).	4a	A
	3. Target Population for Assessment: It is recommended that all patients with diabetes undergo a basic periodontal examination (16, 20). Examine patients for signs of gingival inflammation (such as swelling, bleeding, and redness), tooth mobility, tooth sensitivity, gingival recession or abnormal tooth eruption, persistent halitosis, chewing pain, taste alterations, xerostomia (dry mouth), mucosal changes, dental caries, and candidal infections (26, 27).	5b	A
	4. Assessment Tools: A probe is used to evaluate the periodontal attachment status; for patients with signs of periodontitis, more detailed periodontal charts and plaque indices should be adopted to identify oral problem areas requiring management (17, 24) and evaluate the patient’s preferences for plaque control and their ability to remove plaque (24).	5b	A
Controlling risk factors	5. It is recommended that the glycated hemoglobin (HbA1c) level of patients with diabetes should be ≤ 6.5% (20)	5b	B
	6. It is recommended that patients quit smoking, alleviate emotional stress, and reduce alcohol intake (18, 20, 21).	1b	A
Oral hygiene instruction	7. Dental teams are encouraged to establish a good relationship with patients, understand their oral hygiene needs, demonstrate toothbrushing techniques to them, and develop personalized periodontal care plans based on the patients’ ability to remove dental plaque (22, 24).	1b	A
	8. Selection of toothbrushes: either manual or electric toothbrushes can be used, with small brush heads and medium-textured bristles being preferred. When the bristles fall out or become worn, the toothbrush should be replaced promptly (21, 24).	5b	A
	9. Selection of antibacterial toothpaste: for patients with periodontitis undergoing supportive periodontal care, it is recommended to use products containing chlorhexidine, triclocarban, and stannous fluoride-sodium hexametaphosphate to control gingival inflammation (21).	1a	A
	10. Frequency of toothbrushing: teeth and the gingival margin should be cleaned twice a day (with the last cleaning session performed before bedtime), and each cleaning session should last for at least 2 min (20, 24, 27).	5b	A
	11. Selection and use of interdental cleaning tools: for patients with periodontitis, before toothbrushing, interdental brushes of different sizes are preferred for cleaning the interdental spaces; when in use, the brushes should be closely pressed against the interdental spaces. Meanwhile, dental floss, rubber or elastic cleaning sticks, wooden sticks, and oral irrigators can also be used for interdental cleaning (21, 24).	1a	A
	12. Selection of antibacterial mouthwash: for short-term use, products containing chlorhexidine, essential oils, and cetylpyridinium chloride are recommended to control gingival inflammation (21).	1a	A
Dietary management	13. Carbohydrate Intake: Carbohydrate intake should be dominated by starchy carbohydrates, such as potatoes with skin, whole-wheat bread, rice, and pasta, to obtain more fiber, vitamins, and minerals (20).	5b	B
	14. Protein intake: for protein intake, consume legumes, fish, eggs, meat, and other protein sources. Eat at least 280 grams of fish per week, with one serving being oily fish. Limit the intake of processed meats: sausages, bacon, and cured meats. Encourage the selection of low-fat dairy products and their alternatives (20).	5b	B
	15. Fat intake: for fat intake, reduce the consumption of oils, spreads, and foods high in saturated fat, such as cheese, chocolate, pastries, cakes, and biscuits (20).	5b	B
	16. Dietary fiber intake: for dietary fiber intake, consume at least 5 servings of a variety of fruits and non-starchy vegetables daily. One serving of fruit or vegetables is approximately 80 grams; if dried fruits are chosen as the fruit type, the intake of dried fruits should be 30 grams. If fruits are consumed in the form of fruit juices or Bsmoothies, the total daily amount should be limited to 150 milliliters (20).	5b	B
	17. Beverage recommendations: replace sugary drinks with water, low-fat milk, or sugar-free alternatives (such as tea and coffee). It is recommended to consume 6 to 8 cups of water daily (20).	5b	B
	18. Salt intake: adults should consume no more than 6 grams of salt per day (20).	5b	B
Mechanical intervention	19. Principles of intervention: when the fasting blood glucose level ranges from 4.4 to 7.0 mmol/L and the glycated hemoglobin (HbA1c) level is less than 7.5%, periodontal mechanical intervention can be performed (28).	5b	B
	20. Timing of periodontal intervention: the optimal time for mechanical intervention is recommended to be mid-morning: 1 to 3 h after breakfast and approximately 1.5 h after taking hypoglycemic medications. During the intervention, movements should be as gentle as possible, and the duration should be controlled within 2 h (26, 28).	5b	A
	21. It is recommended to perform mechanical intervention (subgingival scaling and root planing) within 24 h to remove dental calculus, dental plaque, and diseased cementum, thereby effectively controlling periodontal inflammation and improving blood glucose levels (21, 23, 29, 31).	1a	A
	22. After mechanical intervention, chlorhexidine mouthwash should be used twice a day. Once normal toothbrushing is resumed (typically 2 weeks after scaling), brush teeth twice a day (using a manual or electric toothbrush), use dental floss once a day, and quit smoking as appropriate (18).	1c	A
	23. A first-aid kit should be available in the dental clinic, and the kit should contain a blood glucose meter, ultra-rapid-acting insulin, glucagon injection, sugar cubes, and other items (26).	5b	A
Auxiliary methods	24. During mechanical intervention, antibacterial photodynamic therapy (aPDT) can be applied to improve the patient’s periodontal condition (30).	1a	B
	25. During mechanical intervention, it is recommended to appropriately supplement propolis preparations, which can effectively improve blood glucose levels (32).	1a	B
	26. During mechanical intervention, it is recommended to place local antibiotic preparations subgingivally, such as 2% minocycline hydrochloride microspheres, 10% doxycycline hydrochloride sustained-release solution, chlorhexidine periodontal sustained-release agents, and 25% metronidazole gel, to reduce the depth of periodontal pockets and the degree of periodontal attachment loss (17).	1a	B
Follow-up management	27. Interval between visits: based on the outcomes of periodontal treatment and the assessment of dental disease risk, the interval for oral health check-ups shall be determined for each patient, with the interval between clinical visits ranging from 3 to 12 months (19, 21).	5b	A

## 4 Discussion

### 4.1 Comprehensive assessment and early identification of periodontitis signs

Multiple guidelines and expert consensuses (16, 19, 26) clearly state that all patients with diabetes should undergo periodontal examinations. Clinical medical workers should pay attention to the periodontal status in patients’ oral cavities and regard periodontal examination as part of the continuous management of diabetes. However, restricted by the specialization system of various departments, the attention rate and inquiry rate of endocrinologists towards periodontitis during diagnosis and treatment are only 13 and 3.8%, respectively (33). This mainly arises because departments within the modern healthcare system operate relatively independently with well-defined scopes of practice and standardized workflows, and standardized interdisciplinary clinical guidelines and referral mechanisms are lacking. Although the bidirectional relationship between diabetes and periodontitis has been well established, endocrinologists rarely include periodontal assessment in routine diabetes management in clinical practice, and dentists seldom systematically evaluate patients’ diabetic complications prior to treatment. No clear and operable interdisciplinary referral pathway has been established between the two specialties, which further restricts the implementation of integrated care. This consequently results in inadequate awareness of periodontitis among clinicians managing patients with type 2 diabetes. Epidemiological studies (34) have shown that the prevalence of periodontitis gradually increases with the prolongation of patients’ disease course and poor long-term blood glucose control. Moreover, the incidence and severity of diabetes-related complications—such as retinopathy, neuropathy, proteinuria, and cardiovascular diseases—are positively correlated with the severity of periodontitis. Additionally, other studies (35) indicate that compared with diabetic patients without severe periodontitis, those with comorbid severe periodontitis have a threefold increased risk of developing massive albuminuria, end-stage renal disease, and ischemic heart disease. This further underscores the importance of paying attention to the periodontal health of diabetic patients.

Items 1–4 of the evidence cover the assessment tools and specific content for periodontitis in patients with type 2 diabetes mellitus. A simple and rapid basic periodontal examination (BPE) using a probe can be performed on all diabetic patients to assess the presence of gingival bleeding, gingival recession, the status of dental calculus, and other plaque retention factors. Additionally, scores are assigned to identify periodontal problems clearly (36). If the BPE score is 3 or 4, the patient should be referred to the stomatology department. A more detailed 6-point pocket chart and plaque score assessment should be used to evaluate the extent of deep periodontal pockets in the entire dentition, identify oral problem areas requiring management, and avoid missing furcation lesions, thereby providing targeted treatment (20). Meanwhile, the disease course, medication history, and blood glucose control status of diabetic patients should also be documented in detail. Attention should be paid to whether patients are using sulfonylurea drugs or insulin preparations for blood glucose reduction, so as to avoid hypoglycemia during periodontal treatment. If patients have been taking antidepressants or calcium channel blockers for a long time, special attention should be paid to checking for symptoms of xerostomia (dry mouth) and gingival enlargement. This is because such medications are prone to causing overgrowth of gingival tissue, especially when oral hygiene is poor. If patients have been taking cardiovascular medications (such as statins and antihypertensive drugs) for a long time, it is recommended to avoid using preparations containing epinephrine or its derivatives during periodontal treatment to prevent subsequent hyperglycemic events (26). Furthermore, for older adults with diabetes mellitus complicated by multiple comorbidities, in addition to evaluating their systemic disease history and treatment history, it is essential to assess the current control status of their complications. For instance, in older adults with concurrent nephropathy, renal function should be evaluated; periodontal treatment is not advisable if the blood urea nitrogen (BUN) level is ≥ 60 mg/dl and the serum creatinine (Scr) level is ≥ 1.5 mg/L (28). During the diagnosis and treatment of this patient population, enhanced monitoring of clinical symptoms is required. Operatively, the intensity of stimulation should be strictly controlled, and the duration of a single visit should be shortened to reduce the patient’s stress response. If any discomfort occurs, treatment must be immediately discontinued, and a consultation with relevant specialists should be sought for appropriate management. In addition, clinical medical workers should also assess patients’ preferences for interdental plaque control, including the methods used for daily plaque control and the selection of auxiliary tools. This helps determine patients’ ability to remove interdental plaque and set practical tooth-brushing goals for them.

### 4.2 Active prevention to reduce the occurrence of periodontitis

The results of the Fourth National Oral Health Epidemiological Survey show that the prevalence of periodontitis among adults is as high as 62.4% (37). It is recommended that dental professionals and healthcare providers in various clinical departments should actively promote the prevention and treatment technologies for oral diseases to comprehensively improve the level of oral health. However, at present, various oral health care service institutions are treatment-oriented when addressing oral diseases. They neglect the provision of basic oral health education for patients with type 2 diabetes and the enhancement of their health promotion awareness, and lack relevant measures to prevent diseases from the source. Items 5–6 of the evidence focus on the control of risk factors for periodontitis. Diabetes mellitus is a major risk factor for the occurrence and progression of periodontitis. Studies have shown that compared with diabetic patients with well-controlled blood glucose levels or non-diabetic patients, the incidence of periodontitis in patients with poorly controlled blood glucose levels is increased by 86% (38). Meanwhile, the severity of periodontitis is also closely related to poor blood glucose control. Therefore, the guideline (20) suggests that patients should be encouraged to stabilize their blood glucose levels by changing unhealthy lifestyle habits such as quitting smoking, limiting alcohol intake, and maintaining a balanced diet, thereby sustaining the stability of periodontal conditions. Furthermore, it is recommended that diabetic patients maintain their glycated hemoglobin (HbA1c) level at 6.5% or lower. Patients should be informed that an HbA1c level exceeding 7.0% is associated with an increased risk of periodontitis and may exert an adverse effect on the outcomes of subsequent periodontal treatment. However, for patients with multiple comorbidities, the HbA1c target may be appropriately relaxed. The guideline (22) points out that members of the dental team need to listen to patients’ needs, assess the risk factors and adverse behaviors that lead to poor oral health, and provide them with personalized periodontal care plans. Clinical medical staff should select appropriate oral cleaning equipment and usage methods based on patients’ daily tooth-brushing habits and preferences to facilitate long-term adherence to use by patients. Studies by Slot et al. (39) have shown that there is no significant difference between electric toothbrushes and manual toothbrushes in removing plaque from the tooth surface. However, for patients requiring periodontal maintenance, on the premise of considering economic costs, it is recommended to choose electric toothbrushes as an alternative. Items 11–12 of the evidence have a relatively high evidence level and strong practicability, making them easy to promote and apply in clinical practice. Guidelines point out that different cleaning tools such as interdental brushes, dental floss, rubber or elastic cleaning sticks, wooden sticks, and oral irrigators can be selected to maintain the health of interdental gums. Studies have proven (39) that interdental brushes have better cleaning ability than dental floss and are the preferred tool for patients undergoing periodontal maintenance. In addition, it is recommended that under the standardized guidance and demonstration of professionals, interdental brushes of different specifications should be selected based on the size of interdental spaces, and the usage method should be learned. This is to avoid varying degrees of damage to the gums caused by improper operation. The use of chlorhexidine mouthwash can effectively reduce the accumulation of dental plaque and the occurrence of periodontal inflammation; however, its long-term use is prone to adverse reactions such as tooth staining, taste disorders, and oral mucosa changes (24). Therefore, it is recommended that clinicians use this type of mouthwash for a short period of time as appropriate, based on the severity of the patient’s periodontal lesions. The evidence regarding the selection of dietary types is derived from the review of relevant dietary recommendations in the section on managing complications of type 2 diabetes in adults from the NICE Guidelines website (21). Although the level of evidence is not high, it provides a reference for the selection of dietary types and determination of intake amounts.

International research studies have shown that, compared with non-diabetic patients, patients with diabetes complicated by periodontitis have a significantly reduced intake of vegetables, fruits, dairy products, whole grains, as well as vitamins A and C (40). This may be related to the fact that periodontitis impairs patients’ taste, smell, and chewing abilities, leading them to prefer only specific types of food. However, polyphenols and fiber in fruits and vegetables, as well as bioactive compounds in dairy products, can effectively improve periodontal inflammatory biomarkers and reduce alveolar bone loss, thereby delaying the progression of periodontitis (41, 42). Therefore, it is recommended that dietary composition be incorporated as part of routine dental care and guidance. Emphasis should be placed on assessing patients’ basic dietary habits and preferences, and targeted nutritional advice should be provided to maintain the level of oral health.

### 4.3 Timely intervention to slow the progression of periodontitis

Mechanical methods are required to address subgingival calculus and biofilms that are difficult to remove. However, before the intervention, a comprehensive assessment of the patient’s recent blood glucose levels, treatment plan, and presence of cardiovascular and cerebrovascular complications (if any) should be conducted. In clinical practice, however, attention is often only paid to the patient’s fasting blood glucose or random blood glucose, while the assessment of other medical histories is overlooked. Regarding the principles of blood glucose intervention, if the fasting blood glucose (FBG) level is higher than 7.8 mmol/L, the monitoring frequency of glycated hemoglobin (HbA1c) should be increased. If the HbA1c level exceeds 9%, it is recommended to postpone periodontal treatment, with subsequent dental management to be conducted after the blood glucose is stabilized. For diabetic patients complicated by multiple comorbidities or those receiving high-dose insulin therapy, periodontal treatment may also be implemented when their HbA1c level is below 7.5% (43). Meanwhile, studies have shown that for older adults with periodontitis who have a long disease duration, poor blood glucose control, and comorbidities such as hypertension or other cardiovascular and cerebrovascular diseases, it is recommended to conduct screening for vascular lesions before treatment to reduce the incidence of cardiovascular and cerebrovascular complications during the treatment period (44). In terms of selecting the timing for periodontal intervention, the peak effect period of hypoglycemic drugs should be avoided. The 1–3 h after breakfast is a phase when blood glucose rises to its peak and then falls back to a normal level; this time window can avoid the risk of hypoglycemia occurring in the fasting state. Meanwhile, the duration of the intervention should be controlled within 2 h, which can avoid affecting the patient’s normal diet and reduce the occurrence of hypoglycemic events (45). It is necessary to equip dental clinics with first-aid kits. The guideline (43) states that when hypoglycemia occurs in patients with diabetes, 15-20 g of rapidly absorbed carbohydrates, or intravenous glucose should be administered promptly. Therefore, medical staff are recommended to appropriately prepare the items in the first-aid kit according to the actual clinical situation.

The introduction of adjuvant therapeutic modalities has offered diverse clinical strategies for the comprehensive management of periodontitis in diabetic patients. Although antimicrobial photodynamic therapy (aPDT) incurs a relatively higher treatment cost, its clinical efficacy is comparable to that of topically administered antibiotics. Topical antibiotics can effectively improve periodontal probing depth (PD) in the short term (6–9 months) but lack long-term clinical effectiveness. Moreover, their practical application requires consideration of economic costs and drug accessibility (21). Moreover, in practical application, their economic cost and drug accessibility must be taken into consideration. In contrast, as a local treatment modality, aPDT can avoid the potential risks associated with systemic medication, making it particularly suitable for patients with contraindications to antibiotics. Meta-analyses have demonstrated significant heterogeneity in the effects of aPDT on periodontal clinical parameters: there is high consistency in its improvement of bleeding on probing (BOP), while moderate to high heterogeneity exists in its effects on probing depth (PD) and clinical attachment level (CAL) (30). For patients with type 2 diabetes mellitus complicated by mild to moderate chronic periodontitis, the combination of aPDT and NSPT has been shown to reduce glycated hemoglobin (HbA1c) levels from a baseline of 7.85 to 7.24% after 3 months of intervention, exhibiting a marked downward trend compared to the baseline (46). Furthermore, although no definite severe adverse reactions to aPDT have been reported, its clinical application still faces challenges such as the lack of unified standards for treatment protocols and high requirements for operational standardization. Therefore, clinical practice should still take non-surgical periodontal therapy (NSPT) as the core treatment, with aPDT serving as an adjuvant intervention.

Studies have shown that for patients with type 2 diabetes mellitus complicated by periodontitis, daily supplementation with propolis on the basis of receiving non-surgical periodontal therapy (NSPT) results in a significant decrease in glycated hemoglobin (HbA1c) levels from a baseline of 8.73 ± 0.55% to 7.75 ± 0.48% after 24 weeks of intervention (*p* < 0.01) (32). Propolis possesses multiple biological properties including antioxidant, antibacterial, and anti-inflammatory activities, which can assist in regulating glucose metabolism while improving patients’ periodontal conditions. However, current evidence regarding the long-term application safety of propolis, optimal administration regimens (such as dosage and course of treatment), and stratification of suitable populations remains insufficient, leading to significant limitations in its clinical application. In summary, when applying various adjuvant therapeutic modalities in clinical practice, it is necessary to fully weigh their therapeutic advantages and limitations, and formulate personalized periodontal treatment plans considering patients’ specific conditions and clinical practicalities.

### 4.4 Follow-up and dynamic observation of periodontal status

Since all diabetic patients are at risk of developing periodontitis, a stratified follow-up strategy needs to be established. For newly diagnosed type 2 diabetic patients and type 2 diabetic patients without periodontitis, basic periodontal examinations should be conducted annually. For type 2 diabetic patients with comorbid periodontitis, regular oral examinations are required after receiving professional periodontal intervention. International guidelines (22) point out that the interval between oral examinations should be 3 to 12 months, based on the outcomes of periodontal intervention and the assessment of dental disease risk. Studies have shown that after active periodontal intervention, the initial interval between oral follow-up visits is 3 months. During the next follow-up visit, the periodontal bleeding index (Bleeding on Probing, BOP) should be taken into account. If the BOP index is ≤20%, it indicates that the patient’s periodontal status is relatively stable, and the follow-up interval can be appropriately extended to 4–6 months or even longer (47). Meanwhile, close attention should be paid to patients’ glycemic control during periodontal follow-up. Since glycemic control is closely associated with the treatment efficacy and prognosis of periodontitis, focused monitoring of glycemic parameters (such as HbA1c) is recommended in subsequent follow-up to achieve comprehensive management of both glycemic and periodontal conditions.

In addition, based on the international theoretical research on follow-up intervals and with reference to the unique anatomical characteristics of permanent molars in the Chinese population, domestic scholars have proposed a simplified method for determining follow-up intervals using the 2020 version of the periodontal disease risk assessment tool, which is conducive to clinical application (48). This method can flexibly adjust the follow-up interval by evaluating risk factors such as genetics, environment, and systemic diseases, as well as the status of the patient’s own periodontal inflammation. Therefore, clinical workers can develop personalized follow-up plans based on the severity of the patient’s periodontal lesions.

## 5 Summary

This study summarizes the best evidence regarding non-surgical intervention for periodontitis in patients with type 2 diabetes. It provides an evidence-based practice basis for clinical medical staff to manage periodontitis in patients with type 2 diabetes and also offers a reference for formulating personalized periodontal care plans for these patients. Most of the literature included in this study is derived from international research. Due to regional and cultural differences, when translating and applying the evidence, full consideration should be given to the current status of patients with type 2 diabetes and periodontitis in China, and corresponding modifications and improvements should be made to ensure the rational application of the best evidence.

## References

[1] Tonetti MS Greenwell H Kornman KS. Staging and grading of periodontitis: framework and proposal of a new classification and case definition. J Clin Periodontol. (2018) 45:S149–61. doi: 10.1111/jcpe.12945, 29926495

[2] Ding Y-S Wang F Sun J-Y Shao ZW Zou DR Lu JY . Epidemiological survey of periodontal health in patients with type 2 diabetes mellitus of different ages. J Shanghai Jiaotong Univ. (2021) 41:217–22. doi: 10.3969/j.issn.1674-8115.2021.02.014

[3] Joshipura KJ Muñoz-Torres FJ Dye BA Leroux BG Ramírez-Vick M Pérez CM. Longitudinal association between periodontitis and development of diabetes. Diabetes Res Clin Pract. (2018) 141:284–93. doi: 10.1016/j.diabres.2018.04.028, 29679620 PMC6016543

[4] Graziani F Gennai S Solini A Petrini M. A systematic review and meta-analysis of epidemiologic observational evidence on the effect of periodontitis on diabetes an update of the Efp-Aap review. J Clin Periodontol. (2018) 45:167–87. doi: 10.1111/jcpe.12837, 29277926

[5] Shu RNJ. 2018 international classification of periodontal diseases and implant diseases: clinical application of staging and grading of pe-riodontitis. Stomatologiia. (2020) 40:1–6. doi: 10.13591/j.cnki.kqyx.2020.01.001

[6] Meng HX. Periodontology. Beijing: People′s Medical Publishing House (2020).

[7] He RH Lin J Zhu YS Wang HM Lin ZY. Potential profile analysis of health literacy in diabetic periodontitis patients and its correlation with treatment compliance. Chin J Gen Pract. (2025) 23:1492–5. doi: 10.16766/j.cnki.issn.1674-4152.004162

[8] Yang XR He JF. Pathogenesis of diabetic periodontitis and its local drug delivery treatment strategies. Chin J Tissue Eng Res. (2026) 30:2846–57. doi: 10.12307/2026.109

[9] Zhu ZHY Xing WJ Zhou YF Gu Y. The composition of different types of evidence based problems. J Nurses Train. (2017) 32:1991–4. doi: 10.16821/j.cnki.hsjx.2017.21.025

[10] Alper BS Haynes RB. Ebhc pyramid 5.0 for accessing preappraised evidence and guidance. Evid Based Med. (2016) 21:123–5. doi: 10.1136/ebmed-2016-110447, 27325531

[11] Brouwers MC Kho ME Browman GP Burgers JS Cluzeau F Feder G . Agree ii: advancing guideline development, reporting, and evaluation in health care. Prev Med. (2010) 51:421–4. doi: 10.1016/j.ypmed.2010.08.005, 20728466

[12] Foster MJ Shurtz S. Making the critical appraisal for summaries of evidence (case) for evidence-based medicine (Ebm): critical appraisal of summaries of evidence. J Med Libr Assoc. (2013) 101:192–8. doi: 10.3163/1536-5050.101.3.008, 23930089 PMC3738079

[13] Gu Y Zhang HW Zhou YF Xing WJ. JbI evidence-based health center's quality assessment tool for different types of research—the quality evaluation of diagnostic and economic evaluation. J Nurses Train. (2018) 33:701–3. doi: 10.16821/j.cnki.hsjx.2018.08.008

[14] Institute TJB. The Joanna Briggs Institute reviews' manual:2016 edition (2016). Available online at: https://www.joannabriggs.org (Accessed March 18, 2016).

[15] Wang HY. JBI evidence pre-classification and evidence rank system(2014 edition). J Nurses Train. (2015) 30:964–7.

[16] Wexler DJ. Overview of General Medical Care in Nonpregnant Adults with Diabetes Mellitus-UpToDate[EB/OL].(2025-02-19)[2026-03-25]. Available online at: https://www-uptodate-com.edlibproxy.flysheet.com.tw:8443/contents/overview-of-general-medical-care-in-nonpregnant-adults-with-diabetes-mellitus?search=periodontitis&source=search_result&selectedTitle=31%7E118&usage_type=default&display_rank=31

[17] Chow AW. Complications, Diagnosis, and Treatment of Odontogenic Infections - UpToDate[EB/OL].(2024-05-21)[2026-03-25]. Available online at: https://www-uptodate-com.edlibproxy.flysheet.com.tw:8443/contents/complications-diagnosis-and-treatment-of-odontogenic-infections?search=periodontitis&source=search_result&selectedTitle=2%7E118&usage_type=default&display_rank=2

[18] Wilder RS Moretti AJ. Overview of Gingivitis and Periodontitis in Adults - UpToDate[EB/OL].(2023-05-10)[2026-03-25]. Available online at: https://www-uptodate-com.edlibproxy.flysheet.com.tw:8443/contents/overview-of-gingivitis-and-periodontitis-in-adults?search=periodontitis&source=search_result&selectedTitle=1%7E118&usage_type=default&display_rank=1

[19] NICE. Overview | Type 2 Diabetes in Adults: Management | Guidance | NICE[EB/OL]. (2022-06-29)[2026-03-25]. Available online at: https://www.nice.org.uk/guidance/ng28

[20] Health Improvement and Disparities, Department of Health and Social Care, NHS England-and NHS. Improvement.Delivering’ better Oral Health: An Evidence-Based- Toolkit Forprevention[EB/OL].(2021-11-09)[2026-03-25]. Available online at: https://www.gov.uk/government/publications/delivering-better-oral-health-an-evidence-based-toolkit-for-prevention

[21] Sanz M Herrera D Kebschull M Chapple I Jepsen S Beglundh T . Treatment of stage I-III periodontitis-the EFP S3 level clinical practice guideline. J Clin Periodontol. (2020) 47:4–60. doi: 10.1111/jcpe.13290 32383274 PMC7891343

[22] NICE. Overview | Oral Health Promotion: General Dental Practice | Guidance | NICE[EB/OL]. (2018-06-21)[2026-03-25]. Available online at: https://www.nice.org.uk/guidance/ng30.

[23] GIN. Management of Chronic Periodontitis (2nd Edition) | Guidelines International Network(GIN)[EB/OL].[2026-03-25]. Available online at: https://guidelines.ebmportal.com/management-chronic-periodontitis-2nd-edition

[24] Fakheran O. Evidence summary. Periodontal disease (prevention and treatment): Oral hygiene practices. The JBI EBP Database. (2023) JBI-ES-5265-1.

[25] Fong E. Evidence summary. Periodontitis: Treatment The JBI EBP Database. (2022) JBI-ES-2405-2.

[26] Adda G Aimetti M Citterio F Consoli A Di Bartolo P Landi L . Consensus report of the joint workshop of the Italian Society of Diabetology, Italian Society of Periodontology and Implantology, Italian Association of Clinical Diabetologists (SID-SIdP-AMD). Nutrition Metabolism and Cardiovascular Diseases. (2021) 31:2515–25. doi: 10.1016/j.numecd.2021.03.015 34238654

[27] Sanz G Cerillo A Buysschaert M Chapple I Demmer RT Graziani F . Scientific evidence on the links between periodontal diseases and diabetes: Consensus report and guidelines of the joint workshop on periodontal diseases and diabetes by the International Diabetes Federation and the European Federation of Periodontology. J Clin Periodontol. (2018) 45:138–49. doi: 10.1111/jcpe.12808 29280174

[28] Society Of Periodontology CSA. Consensus of Chinese experts on diagnosis of severe periodontitis and treatment principles of periodontitis in special population. Chinese Journal of Stomatology. (2017) 52:67–71. doi: 10.3760/cma.j.issn.1002-0098.2017.02.002 28253577

[29] Corbella S Alberti A Donos N Morandi B Ercal P Francetti L . Efficacy of different protocols of non-surgical periodontal therapy in patients with type 2 diabetes: a systematic review and meta-analysis. J Periodontal Res. (2025) 60:417–37. doi: 10.1111/jre.13327 39343708 PMC12186468

[30] JR DSPGB Abreu LG Costa FO Cota LOM Esteves-Lima RP. The effect of antimicrobial photodynamic therapy adjunct to non-surgical periodontal therapy on the treatment of periodontitis in individuals with type 2 diabetes mellitus: a systematic review and meta-analysis. Photodiagn Photodyn Ther. (2023):42. doi: 10.1016/j.pdpdt.2023.103573 37062511

[31] Oliveira VB Costa FWG Haas AN JR MRM Rêgo RO. Effect of subgingival periodontal therapy on glycaemic control in type 2 diabetes patients: Meta-analysis and meta-regression of 6-month follow-up randomized clinical trials. J Clin Periodontol. (2023) 50:1123–37. doi: 10.1111/jcpe.13830 37257917

[32] Araújo EG De Oliveira D Martins CC Stefani CM. Efficacy of antioxidant supplementation to non-surgical periodontal therapy on metabolic control in type 2 diabetes patients: a network Meta-analysis. Antioxidants. (2022) 11. doi: 10.3390/antiox11040621 PMC9031448 35453306

[33] Liu Y Wu M Li Y Cong DD Zhong HY Guo LX. A survey on the cognition of endocrinologists and type 2 diabetes patients regarding the relationship between diabetes and periodontitis. Chinese Journal of Diabetes Mellitus. (2012) 7:58–62.

[34] DingY S Shao ZW Lin ZJ Zou DR. Periodontal health survey and analysis of influencing factors in 916 patients with type 2 diabetes. Shanghai Journal of Stomatology. (2022) 31:173–7. doi: 10.19439/j.sjos.2022.02.011 36110075

[35] Preshaw PM Alba AL Herrera D Jepsen S Konstantinidis A Makrilakis K . Periodontitis and diabetes: a two-way relationship. Diabetologia. (2012) 55:21–31. doi: 10.1007/s00125-011-2342-y 22057194 PMC3228943

[36] O Periodontology. B S. Basic Periodontal Examination (BPE) [J/OL] Available online at: https://www.bsperio.org.uk

[37] W X. Report of the Fourth National Oral Health Survey [M]. Beijing: People′s Medical Publishing House. (2018) 106–113.

[38] Alwithanani N. Periodontal diseases and diabetes mellitus: a systematic review. Journal of Pharmacy and Bioallied Sciences. (2023) 15:S54–63. doi: 10.4103/jpbs.jpbs_515_22 37654263 PMC10466651

[39] Slot DE Valkenburg C Van Der Weijden GA. Mechanical plaque removal of periodontal maintenance patients: a systematic review and network meta-analysis. J Clin Periodontol. (2020) 47:107–24. doi: 10.1111/jcpe.13275 32716118

[40] Basu A Richardson LA Carlos A Abubakr NH Weltman RL Ebersole JL. The associations of Cardiometabolic and dietary variables with clinical periodontitis in adults with and without type 2 diabetes: a cross-sectional study. Nutrients. (2023) 16. doi: 10.3390/nu16010081 PMC10780717 38201914

[41] Cooper AJ Forouhi NG Ye Z Buijsse B Arriola L Balkau B . Fruit and vegetable intake and type 2 diabetes: EPIC-InterAct prospective study and meta-analysis. Eur J Clin Nutr. (2012) 66:1082–92. doi: 10.1038/ejcn.2012.85 22854878 PMC3652306

[42] Dodington DW Fritz PC Sullivan PJ Ward WE. Higher intakes of fruits and vegetables, β-carotene, vitamin C, α-tocopherol, EPA, and DHA are positively associated with periodontal healing after nonsurgical periodontal therapy in nonsmokers but not in smokers. J Nutr. (2015) 145:2512–9. doi: 10.3945/jn.115.211524 26423734

[43] Society CD. Guideline for the prevention and treatment of type 2 diabetes mellitus in China (2020 edition). Chinese Journal of Diabetes Mellitus. (2021) 13:315–409. doi: 10.3760/cma.j.cn115791-20210221-00095

[44] Shi XXGJH Ren XY. Diagnosis and treatment strategy of periodontitis with diabetes. Chinese Journal of Stomatology. (2023) 58:615–20. doi: 10.3760/cma.j.cn112144-20230207-00029 37272009

[45] Meng XX. Chinese Guidelines for the Prevention and Treatment of Periodontal Diseases [M]. Beijing: People′s Medical Publishing House (2015).

[46] Mirza S Khan AA Al-Kheraif AA Khan SZ Shafqat SS. Efficacy of adjunctive photodynamic therapy on the clinical periodontal, HbA1c and advanced glycation end product levels among mild to moderate chronic periodontal disease patients with type 2 diabetes mellitus: a randomized controlled clinical trial. Photodiagn Photodyn Ther. (2019) 28:177–82. doi: 10.1016/j.pdpdt.2019.08.003 31394300

[47] Ramseier CA Nydegger M Walter C Fischer G Sculean A Lang NP . Time between recall visits and residual probing depths predict long-term stability in patients enrolled in supportive periodontal therapy. J Clin Periodontol. (2019) 46:218–30. doi: 10.1111/jcpe.13041 30499586

[48] Shao JL Yu Y Lu CX Ge SH. Introduction and interpretation of the European federation of periodontology S3 level clinical practice guideline for treatment of periodontitis. Chinese Journal of Stomatology. (2022) 57:1202–8. doi: 10.3760/cma.j.cn112144-20220719-00394 36509519

