# Supplementary material for: Summary of the best evidence for non-surgical intervention in periodontitis among patients with type 2 diabetes mellitus
Source: Front Public Health. 2026 Apr 20;14:1792923. doi: 10.3389/fpubh.2026.1792923 (PMC13174932; doi:10.3389/fpubh.2026.1792923)
Supplement: Supplementary file 1 [file Table_1.doc]

| Search databases and websites | Search formula | Number | Screenshot Evidence |
| --- | --- | --- | --- |
| Uptodate | “periodontitis” | 43 | 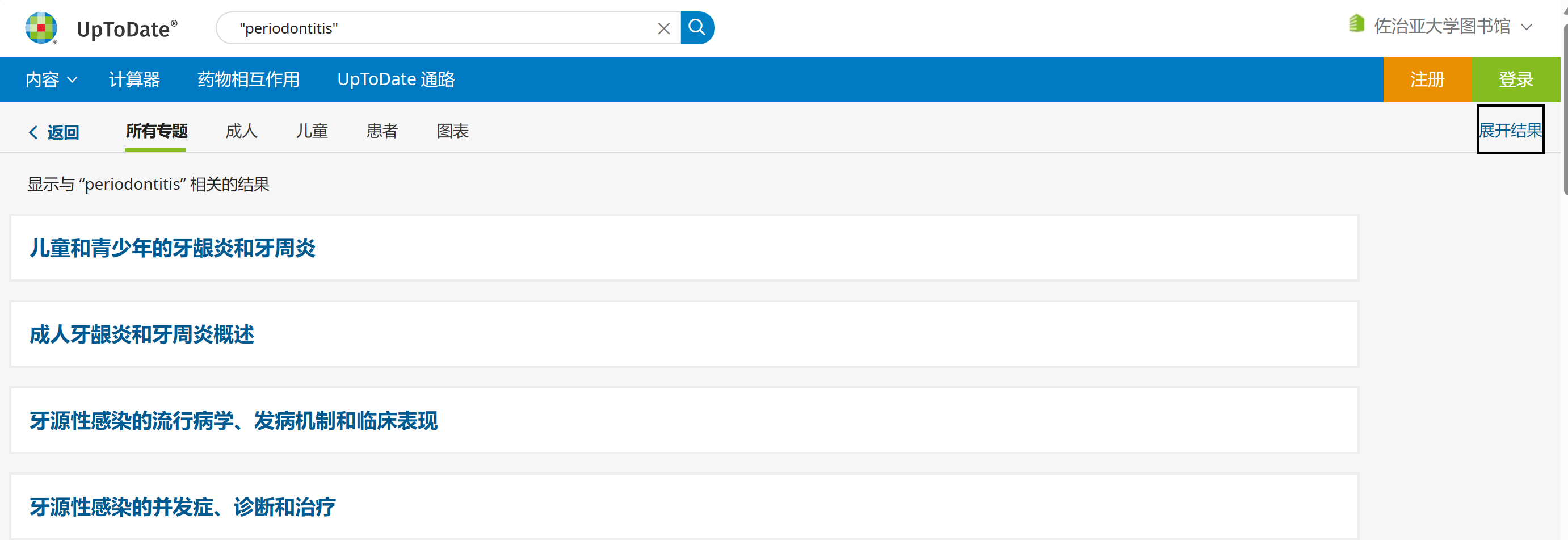 |
| BMJ | “periodontitis” or “diabetes” | 5 | 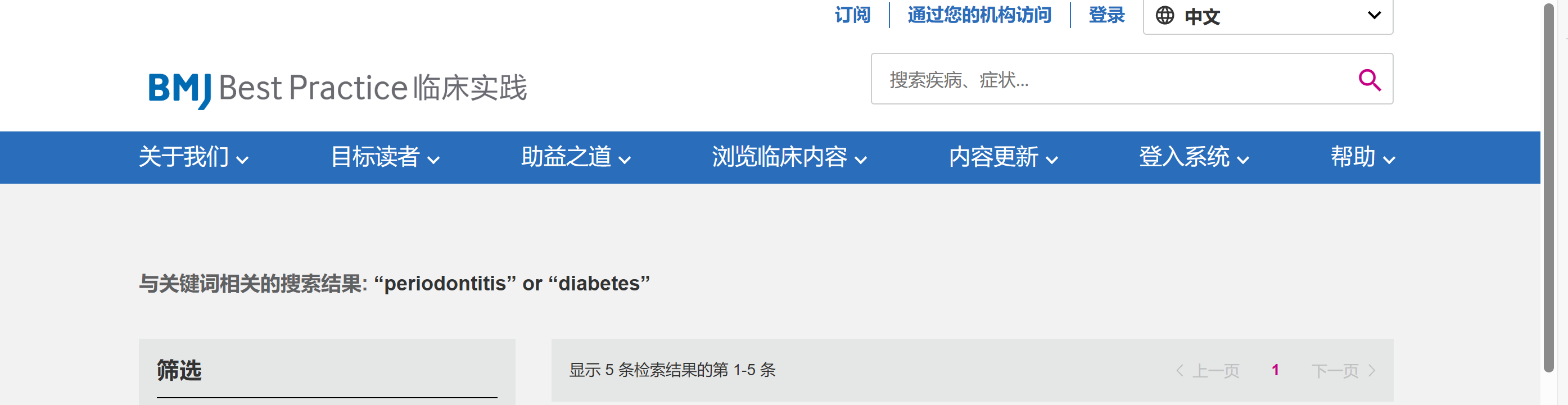 |
| JBI | #1: (periodont* or oral health or oral hygiene or oral care).m_titl.  #2 type 2 diabetes mellitus.m_titl.  #3: #1 and #2  #4 (prevention or management or treatment or evaluat* or assess*).m_titl  #5 #3 and #4 | 30 | 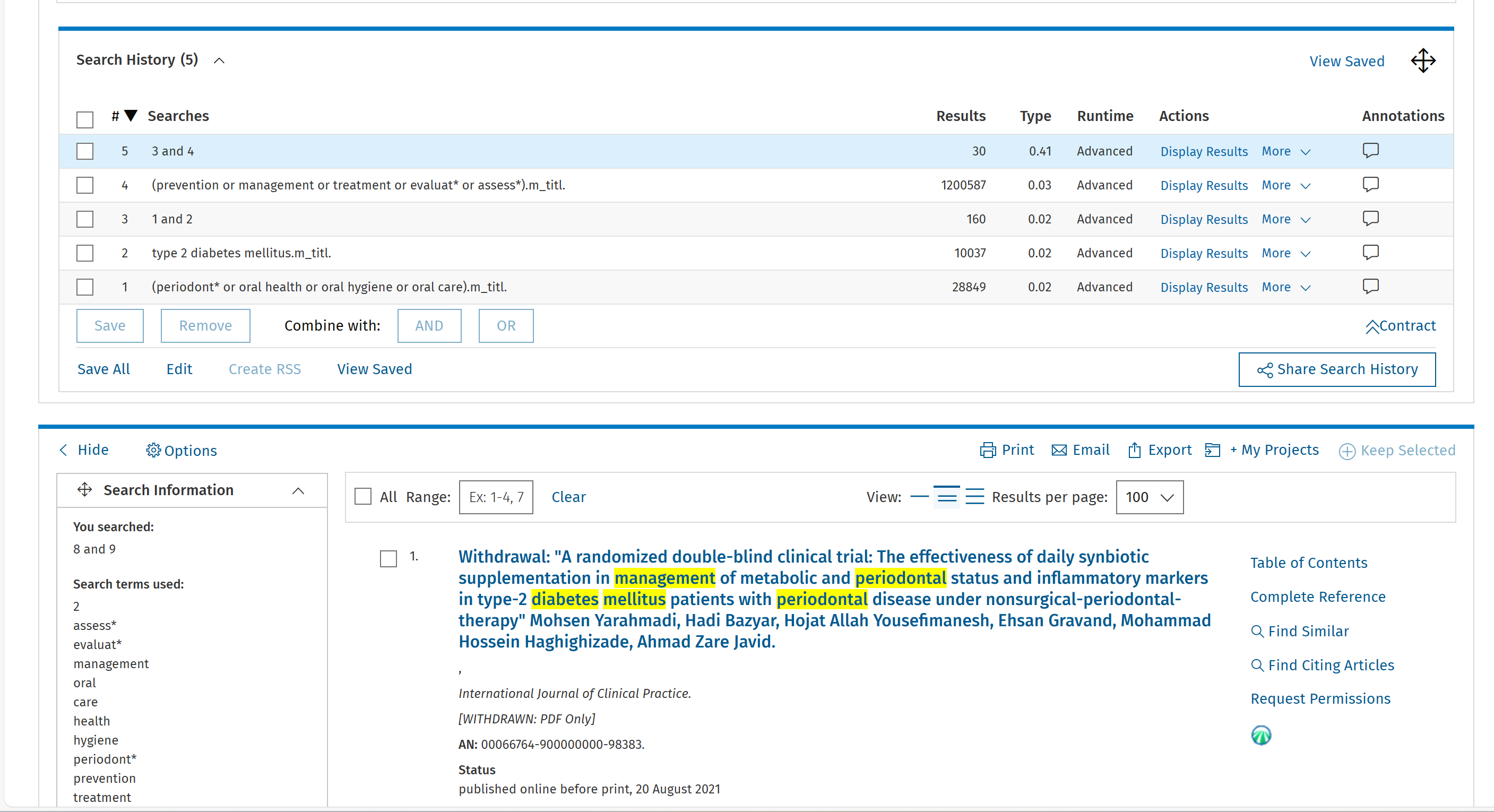 |
| NICE | periodontitis | 7 | 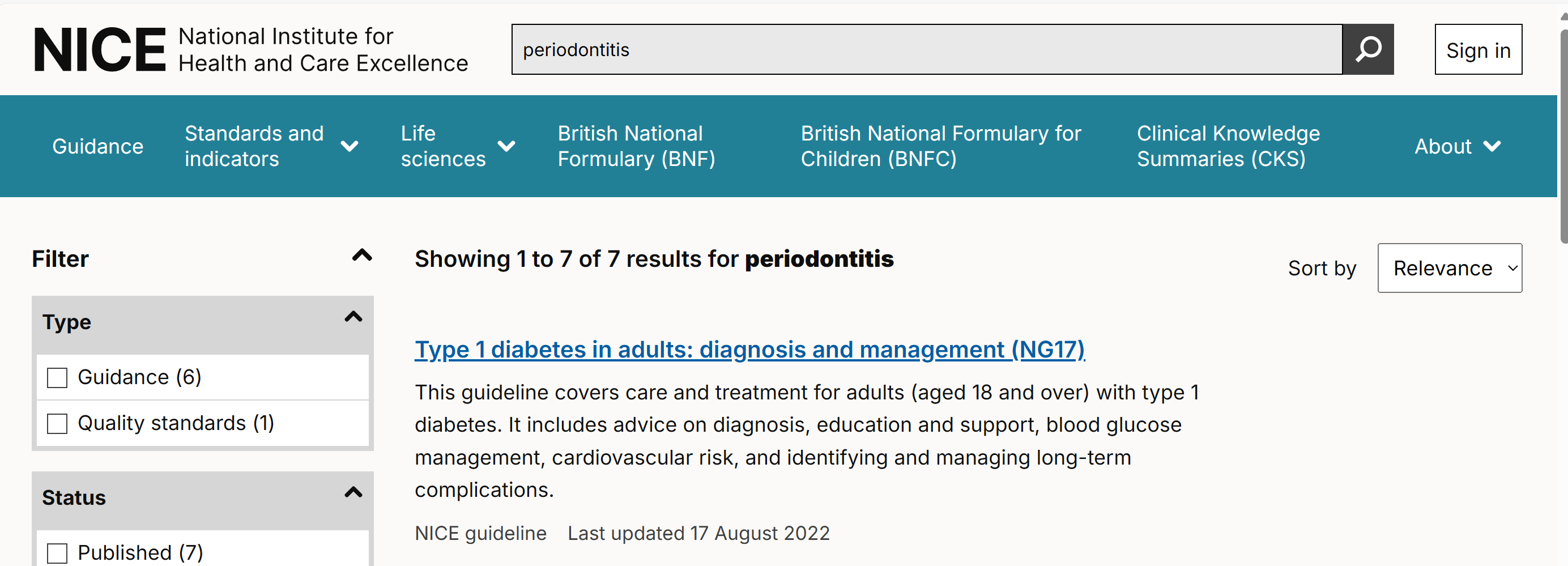 |
| RNAO | Periodontitis | 0 |  |
| GIN  Guidelines International Network (GIN) | Periodontitis | 12 | 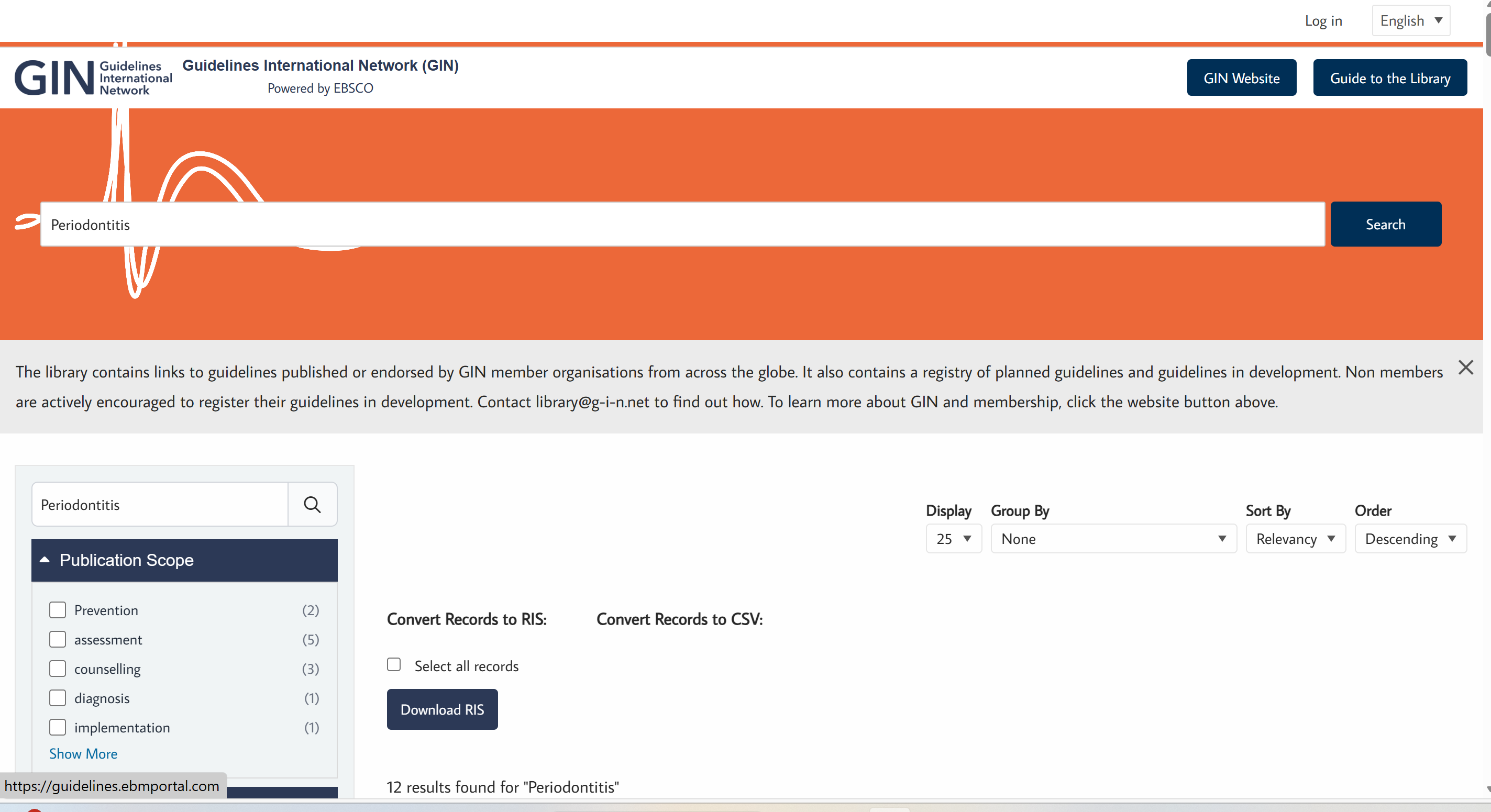 |
| Canadian Medical Association （CMA） | Periodontitis | 3 | 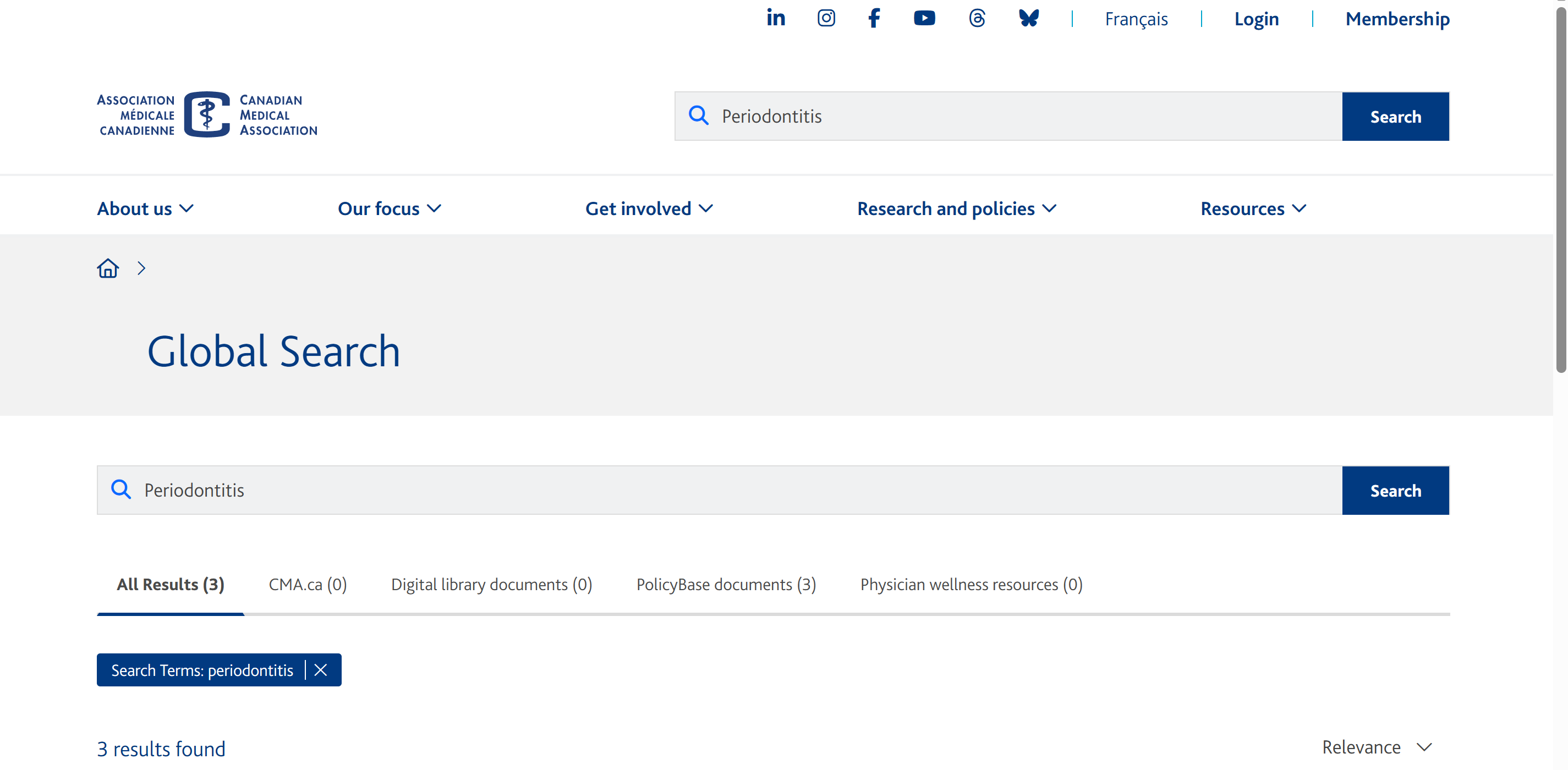 |
| NZGG | Periodontitis | 0 |  |
| Chinese Medical Knowledge Database | 牙周炎 | 6 | 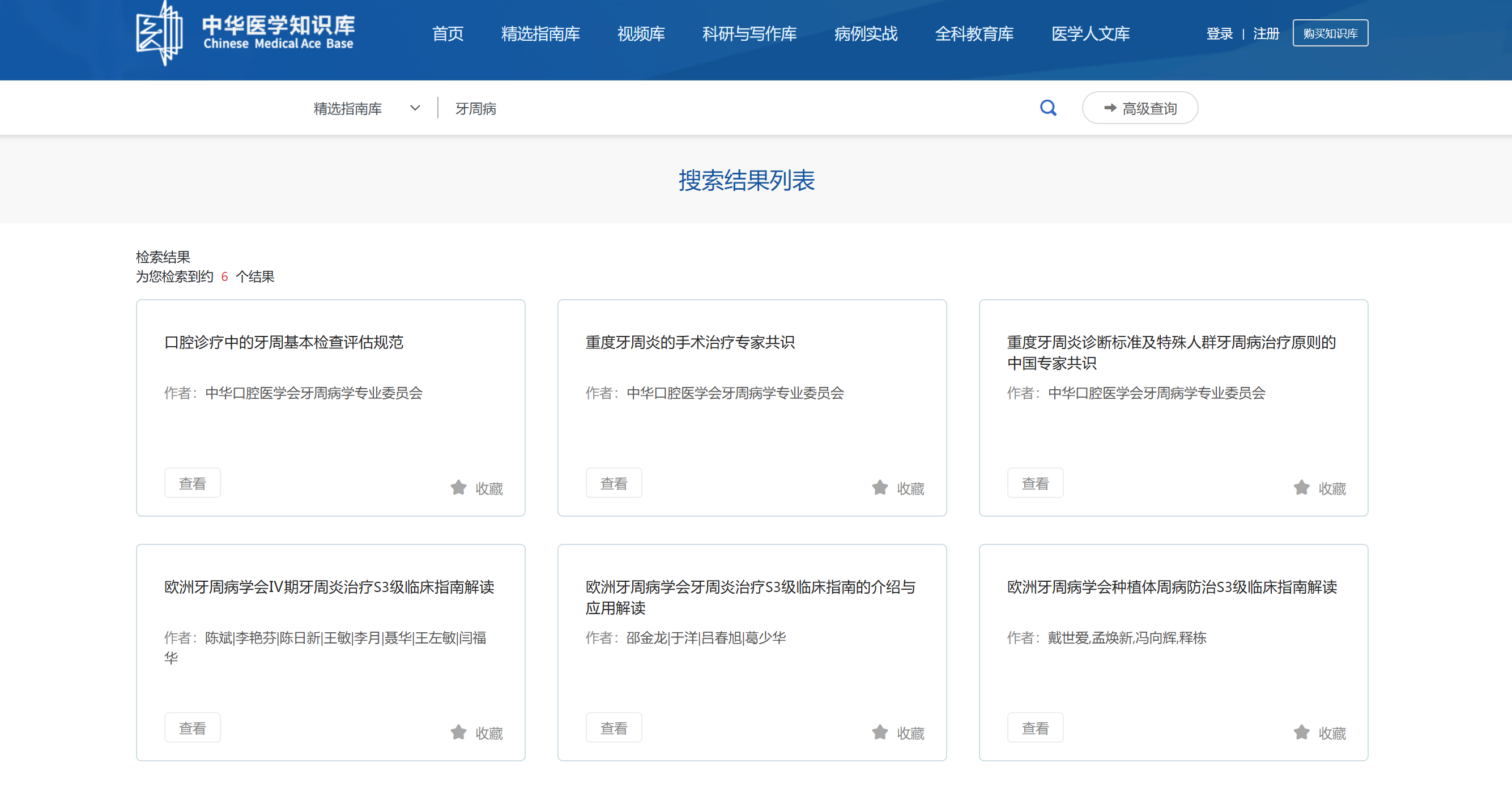 |
| Yimaitong Guidelines Network | 牙周炎 | 444 | 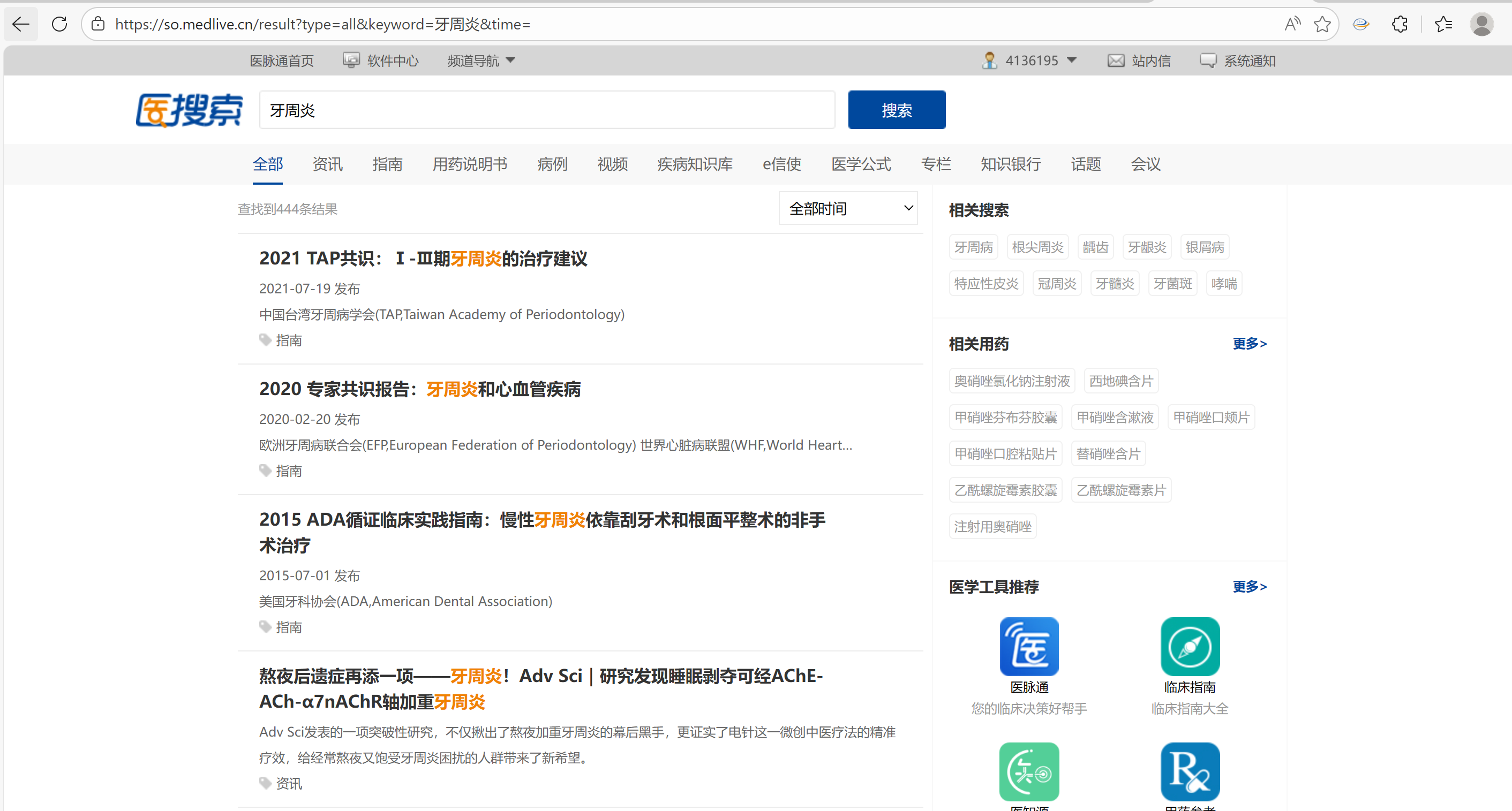 |
| Chinese Stomatological Association (CSA), | 指南 | 33 | 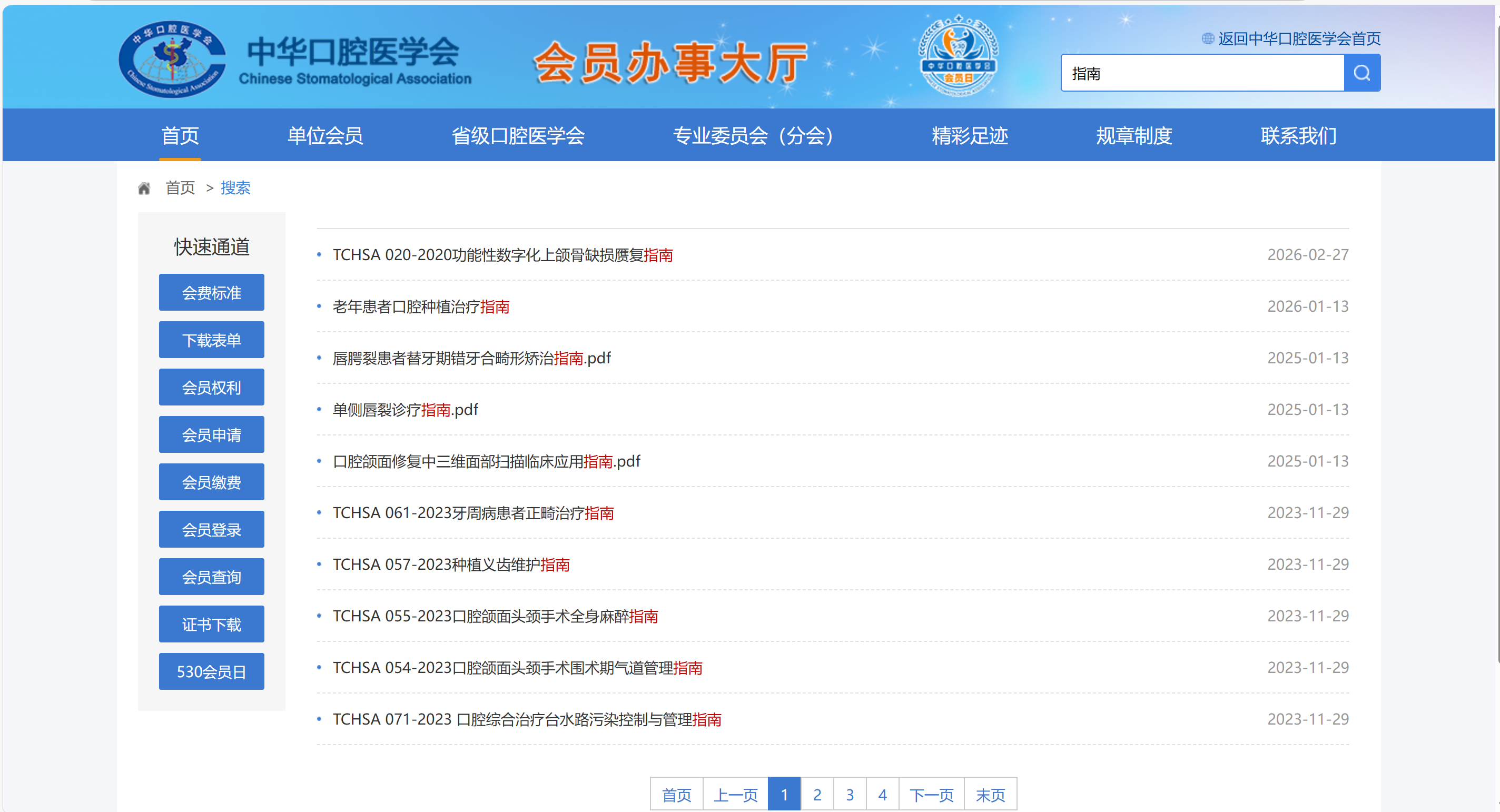 |
| World Dental Federation (FDI), | 指南 | 0 |  |
| American Dental Association (ADA) | periodontitis guideline | 12 | 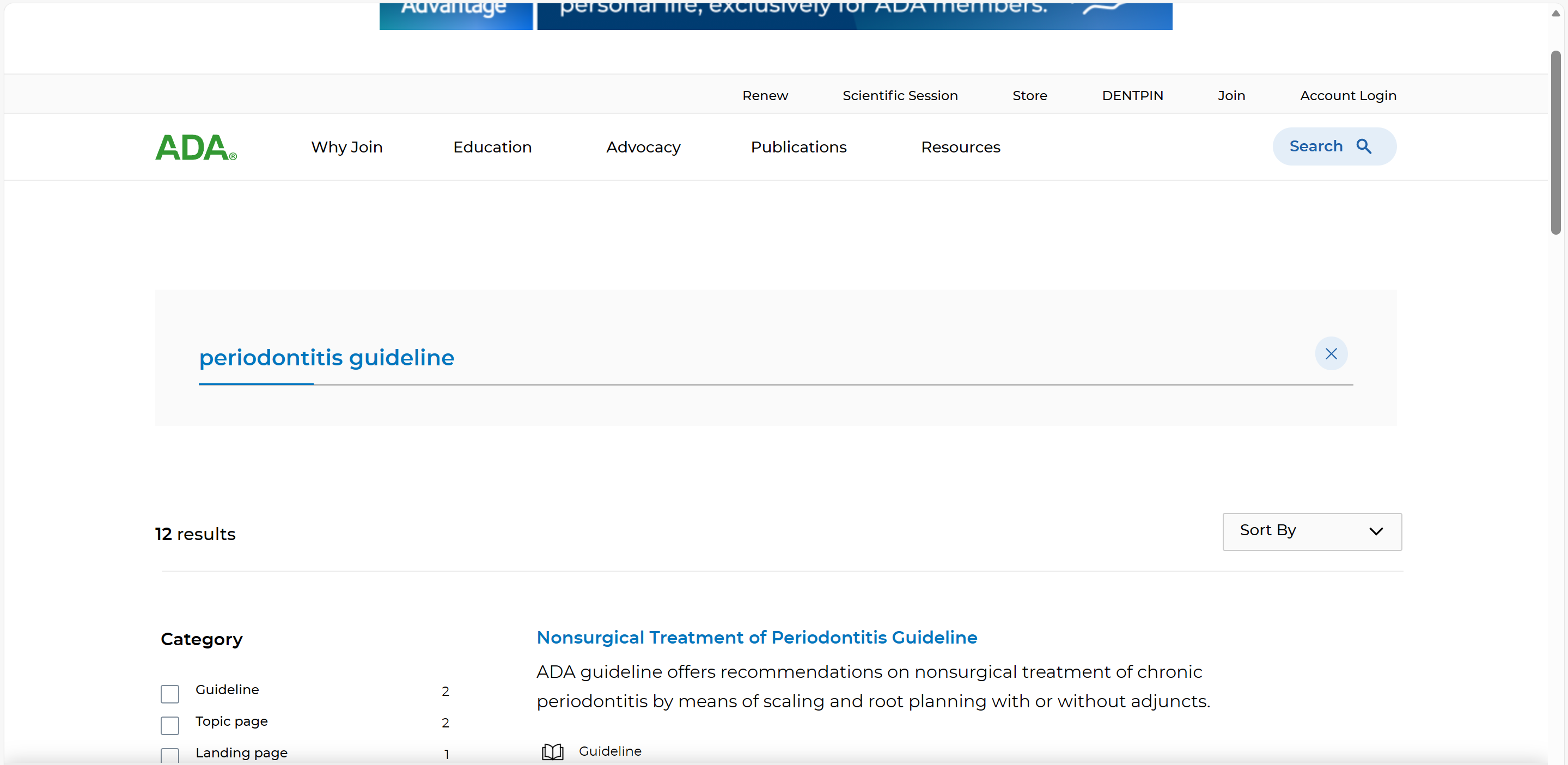 |
| International Association for Dental Research (IADR) | periodontitis guideline | 0 |  |
| Cochrane Library | #1 periodont* in Cochrane Reviews, Trials 20906  #2 MeSH descriptor: [Periodontitis] explode all trees 4224  #3 type 2 diabetes mellitus in Cochrane Reviews, Trials 78148  #4 #1 OR #2 20943  #5 #3 AND #4 651  #6 oral health OR oral hygiene OR oral care OR prevention OR management OR treatment OR evaluat* OR assess* in Cochrane Reviews, Trials 1728193  #7 MeSH descriptor: [Oral Health] explode all trees 881  #8 #6 OR #7 1728193  #9 #5 AND #8 in Cochrane Reviews 18 | 18 | 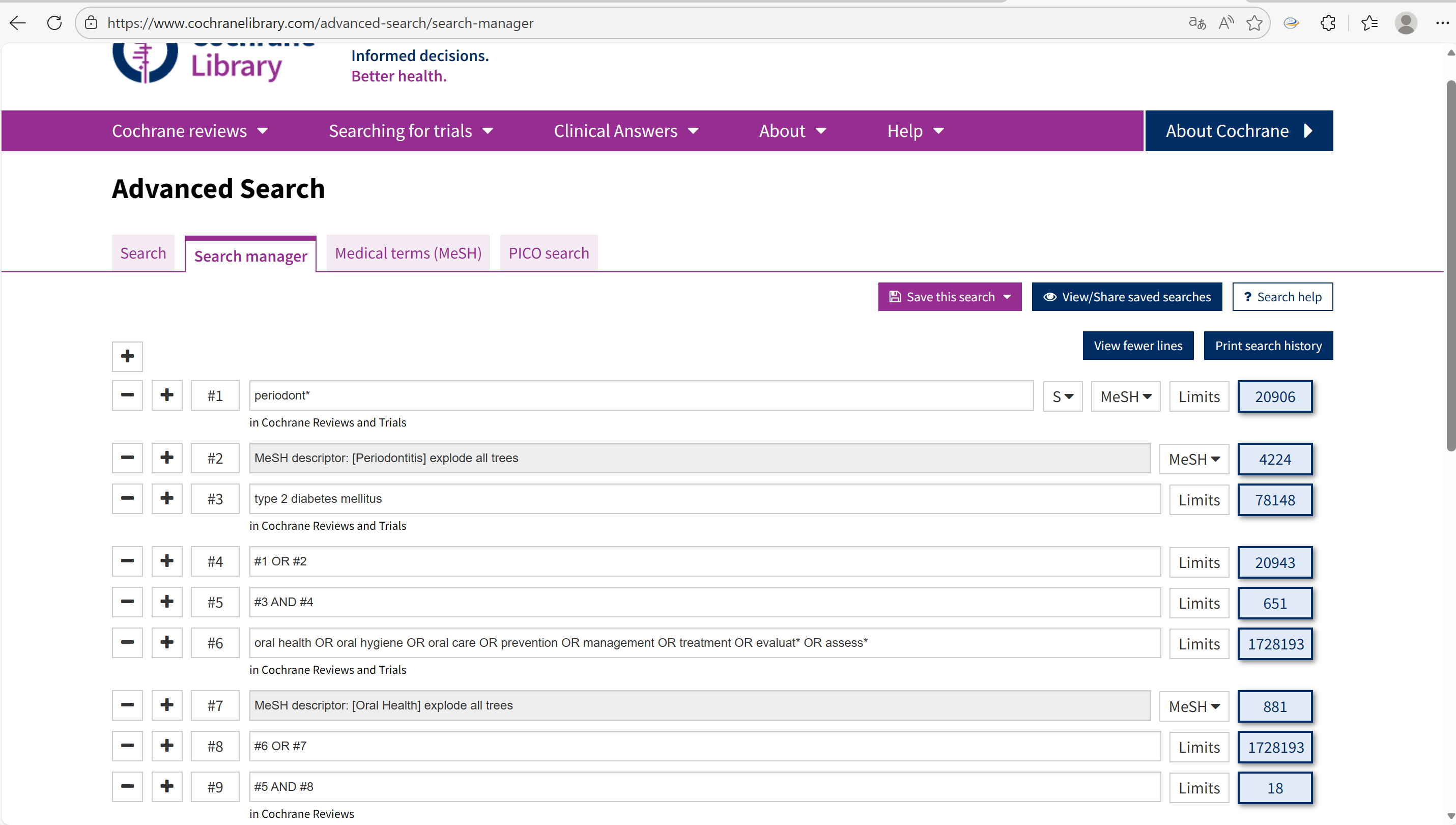  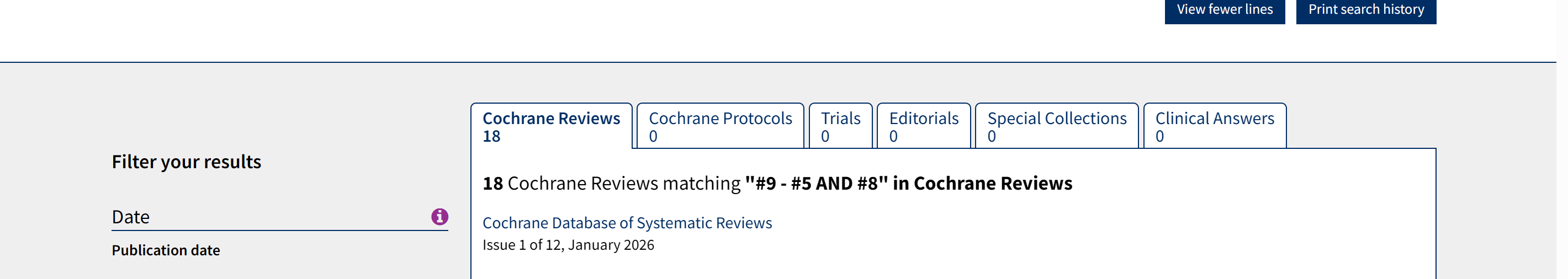 |
| CINAHL | #1 periodont* [All Fields]  #2 type 2 diabetes mellitus [All Fields]  #3 #1 and #2  #4 (oral health OR oral hygiene OR oral care OR prevention OR management OR treatment OR evaluat* OR assess*)[All Fields]  #5 (best practice OR guideline OR evidence summar* OR consensus OR expert opinion OR systematic review OR Meta analysis)[All Fields]  #6 #3 and #4 and # 5 | 76 | 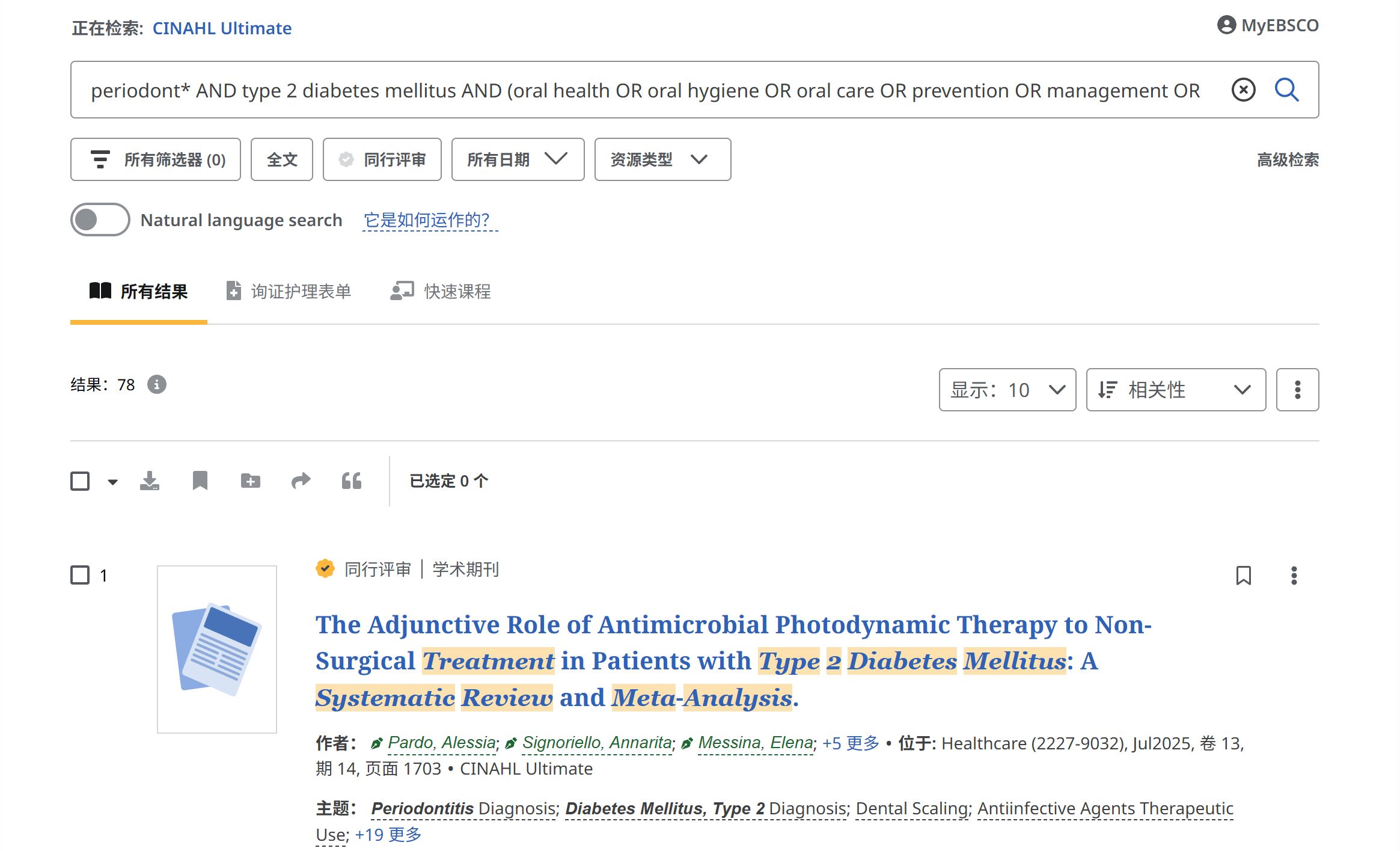 |
| Pubmed | #1 "periodont*"[All Fields] OR "periodontitis"[MeSH Terms]  #2 "diabetes mellitus, type 2"[MeSH Terms] OR "type 2 diabetes mellitus"[All Fields]  #3 ("periodont*"[All Fields] OR "periodontitis"[MeSH Terms]) AND ("diabetes mellitus, type 2"[MeSH Terms] OR "type 2 diabetes mellitus"[All Fields])  #4 ((((((((oral health[All Fields]) OR (oral hygiene[All Fields])) OR (oral care[All Fields])) OR (prevention[All Fields])) OR (management[All Fields])) OR (treatment[All Fields])) OR (evaluat*[All Fields])) OR (assess*[All Fields])) OR (Oral Health[MeSH Terms])  #5 ((((((best practice[All Fields]) OR (guideline[All Fields])) OR (evidence summar*[All Fields])) OR (consensus[All Fields])) OR (expert opinion[All Fields])) OR (systematic review[All Fields])) OR (Meta analysis[All Fields])  #3 AND #4 AND #5 | 133 | 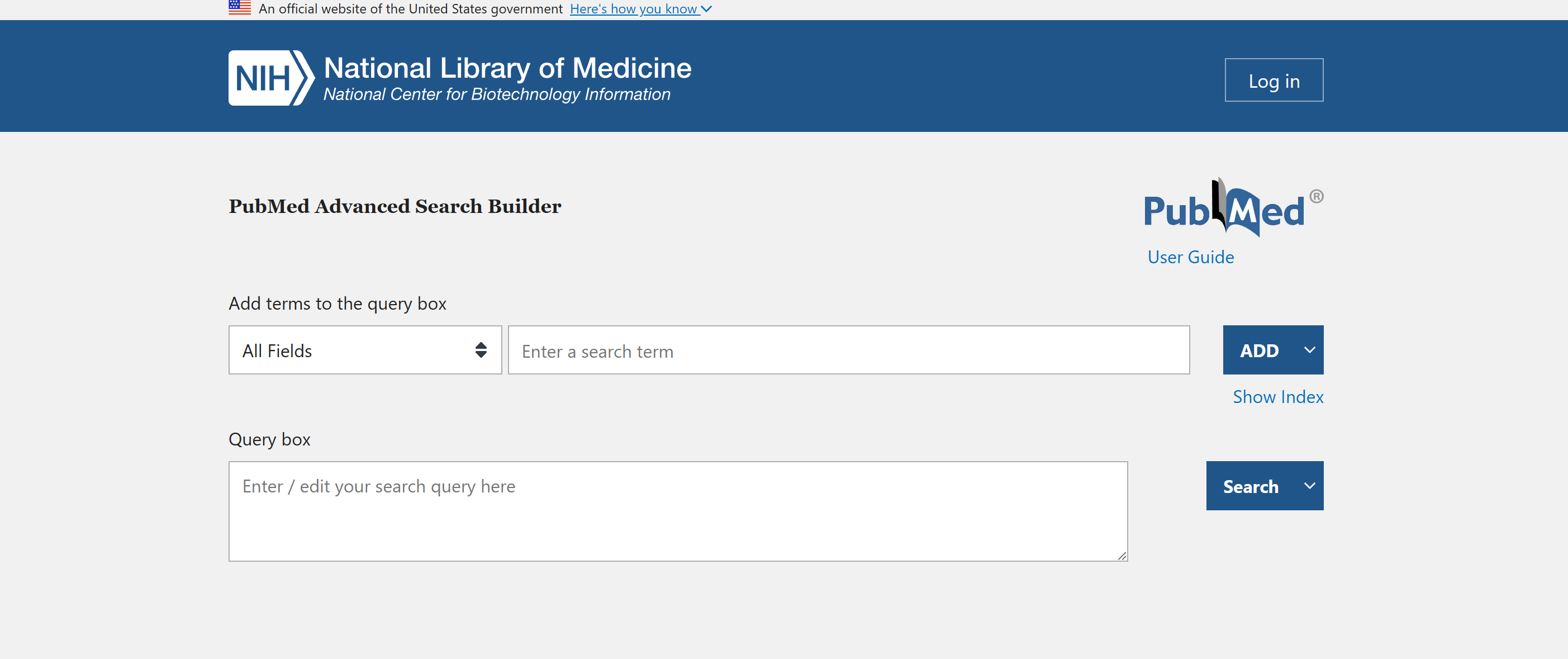  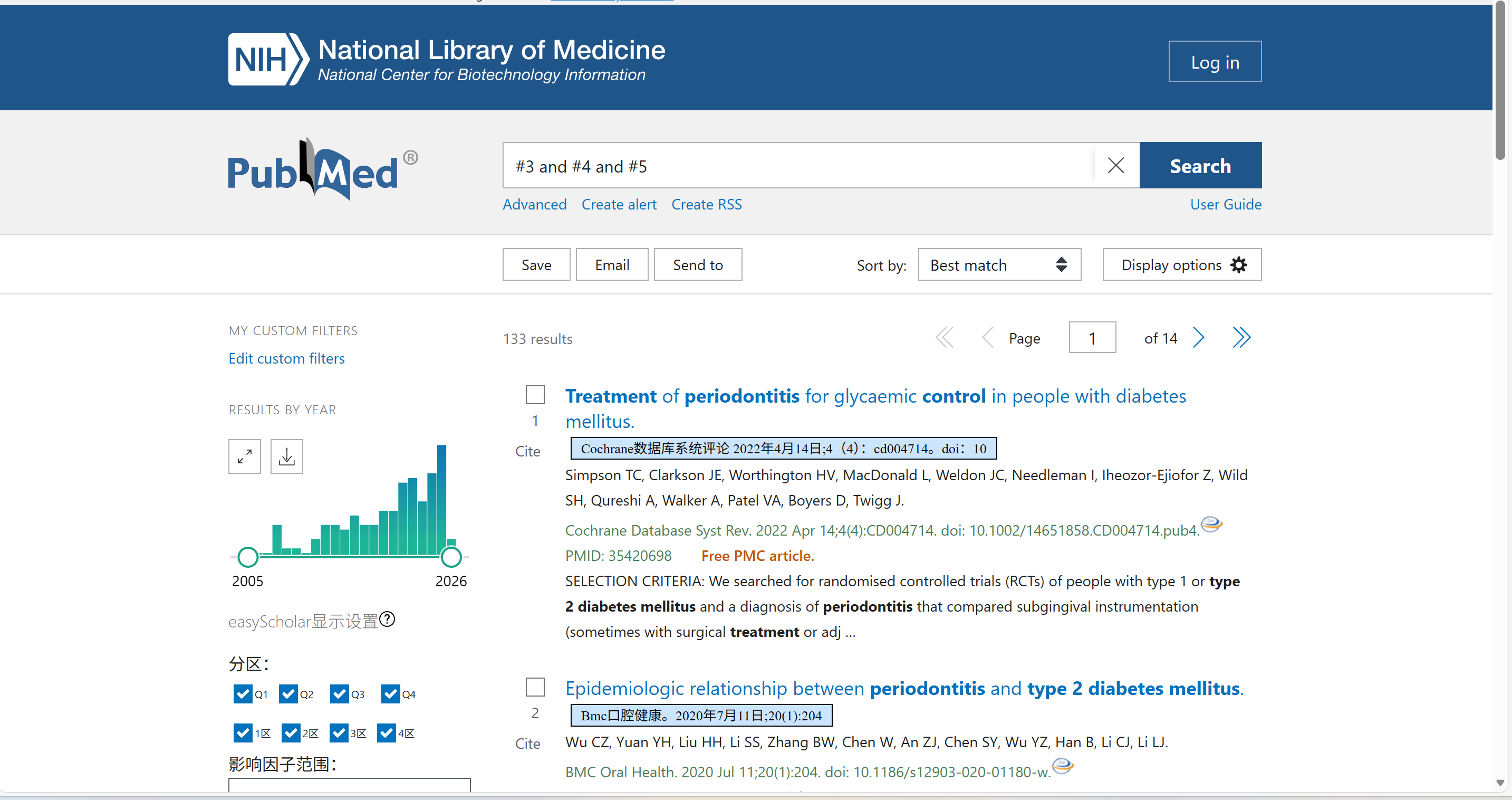 |
| Embase | #10 #5 AND #8 AND #9 46  #9#6 OR #7 17210125  #8'best practice':ab,ti OR 'guideline':ab,ti OR 'evidence summar*':ab,ti OR 'consensus':ab,ti OR 'expert opinion':ab,ti OR 'systematic review':ab,ti OR 'meta analysis':ab,ti 1039127  #7'oral health':ab,ti OR 'oral hygiene':ab,ti OR 'oral care':ab,ti OR 'prevention':ab,ti OR management:ab,ti OR treatment:ab,ti OR evaluat*:ab,ti OR assess*:ab,ti 17201478  #6'mouth hygiene'/exp 35578  #5#1 AND #4 736  #4#2 OR #3119875  #3periodont*:ab,ti101032  #2'periodontitis'/exp59768  #1'type 2 diabetes mellitus':ab,ti  98852 | 46 | 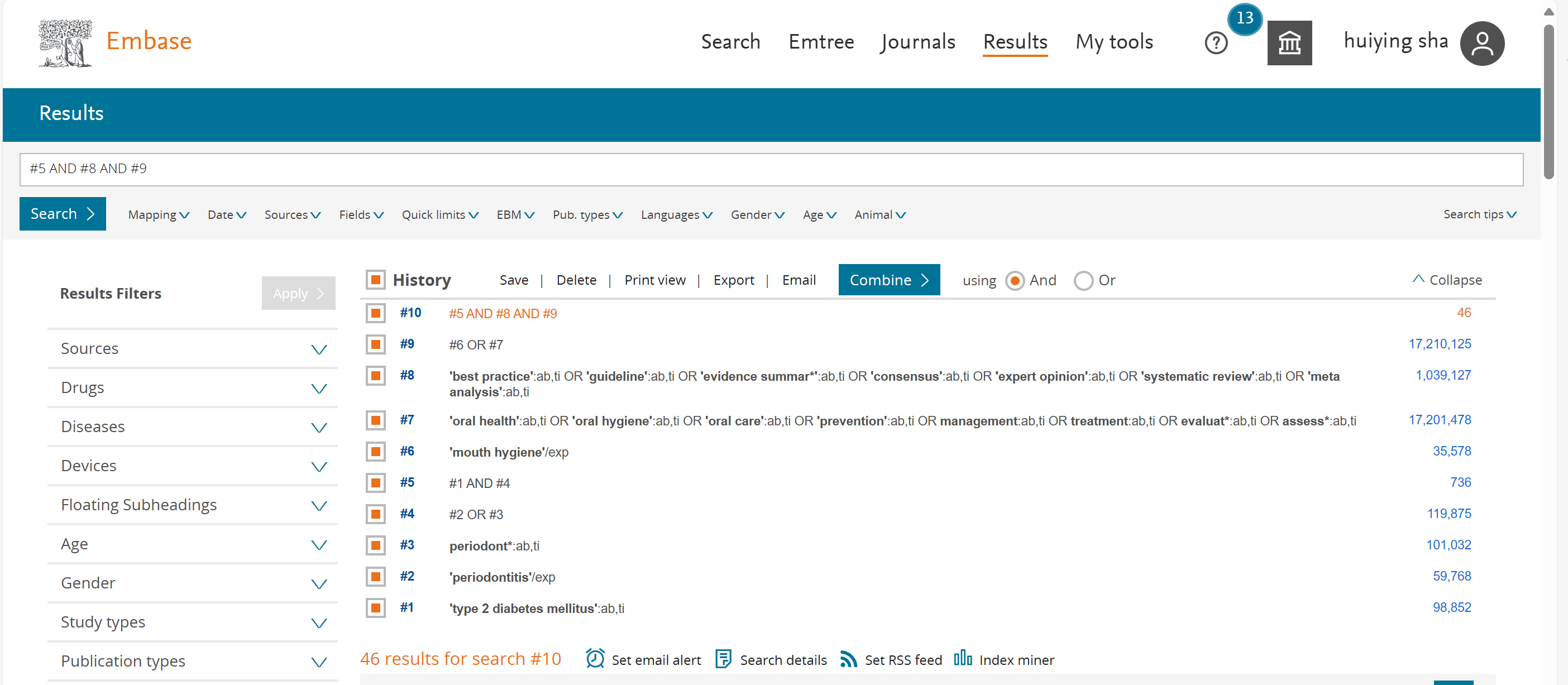 |
| China National Knowledge Infrastructure (CNKI) | （篇关键词：牙周炎 + 牙周病+ 2型糖尿病）AND （篇关键词： 口腔卫生 + 口腔保健+ 口腔健康 +口腔护理+ 预防 + 管理 + 治疗 + 评估）AND（篇关键词：临床实践 + 指南 + 证据总结 + 共识 + 专家 + 系统评价 + 系统综述 + 系统回顾 + 荟萃分析 + Meta分析） | 385 | 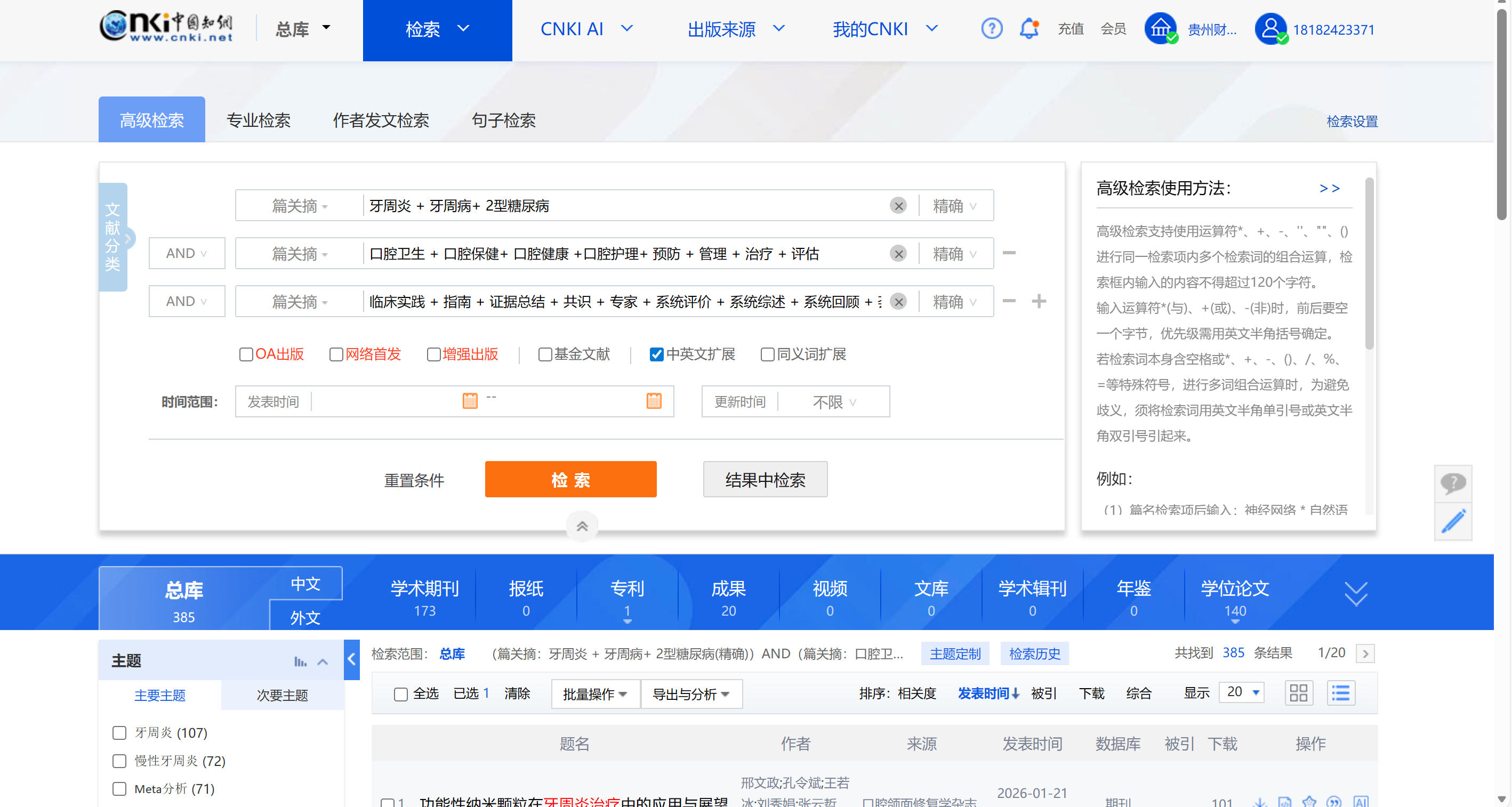 |
| Wanfang Database | （全部：牙周炎 OR 牙周病 OR 2型糖尿病）AND（全部：口腔卫生 OR 口腔保健 OR 口腔健康 OR 口腔护理 ）AND（全部：临床实践 OR 指南 OR 证据总结 OR 共识 OR 专家 OR 系统评价 OR 系统综述 OR 系统回顾 OR 荟萃分析 OR Meta分析） | 722 | 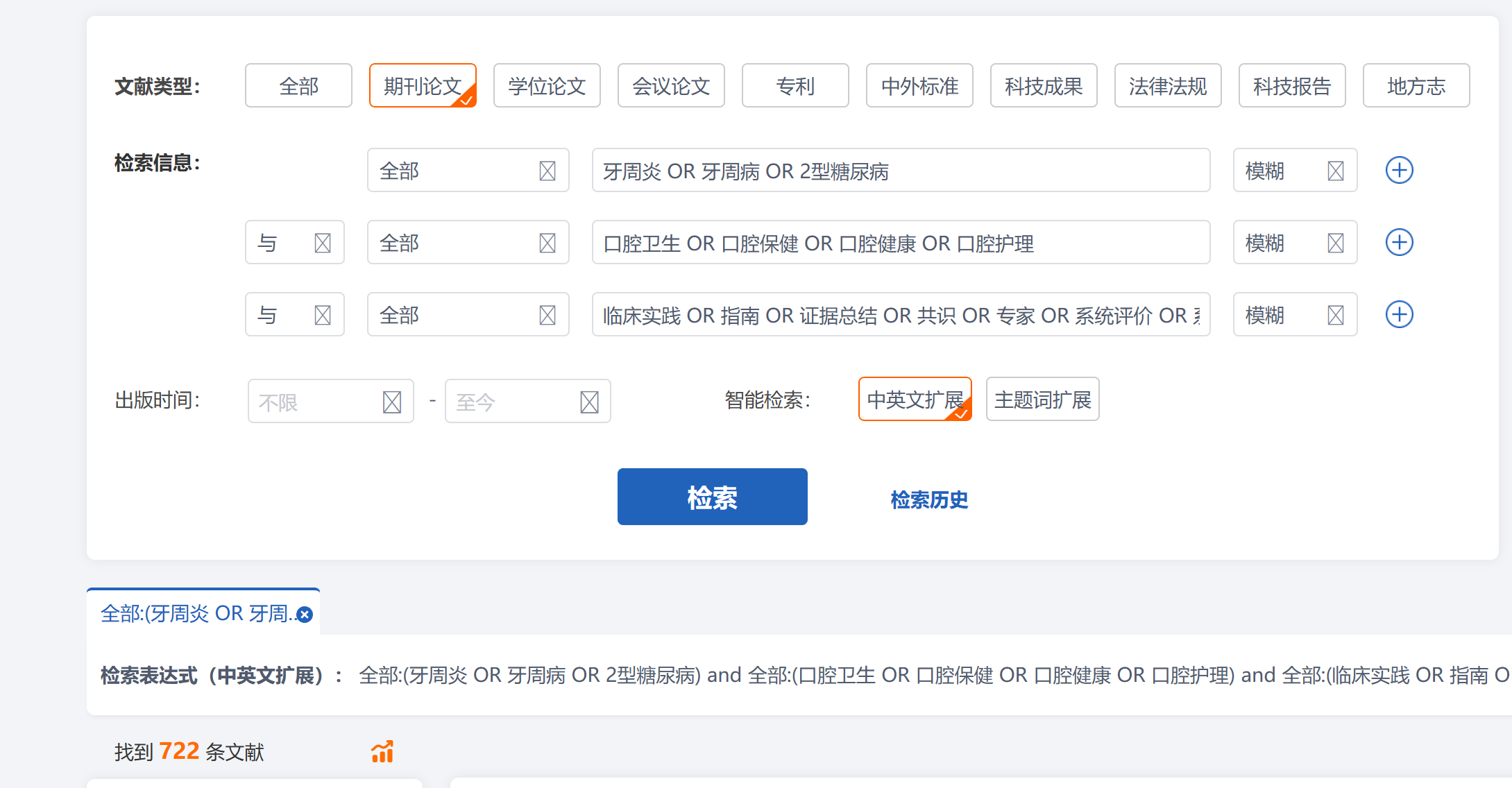 |
