# Supplementary material for: Summary of the best evidence for non-surgical intervention in periodontitis among patients with type 2 diabetes mellitus
Source: Front Public Health. 2026 Apr 20;14:1792923. doi: 10.3389/fpubh.2026.1792923 (PMC13174932; doi:10.3389/fpubh.2026.1792923)
Supplement: Supplementary file 2 [file Table_2.docx]

|  | Literature sources | Evidence extracted from the literature |
| --- | --- | --- |
| 16 | UpToDate-Overview of general medical care in nonpregnant adults with diabetes mellitus：  非妊娠糖尿病成人患者的一般医疗管理概述 | 1、推荐所有糖尿病患者每年接受口腔检查。 |
| 17 | UpToDate:Complications, diagnosis, and treatment of odontogenic infections牙源性感染的并发症、诊断和治疗(牙源性感染包括龋齿和牙周病) | **2、成人非重度牙周炎**−对于非重度成人牙周炎(2017年牙周病和种植体周围病国际分类根据病情的严重程度及管理复杂性归为Ⅰ期或Ⅱ期[[33](https://www-uptodate-cn-s--zhe.cnu100.rbltsg.top/contents/zh-Hans/complications-diagnosis-and-treatment-of-odontogenic-infections/abstract/33)])，可在刮治+根面平整的同时，在龈下放置局部用抗生素制剂、释放一定量的抗生素，以减少牙周袋深度。据报道，这种局部抗生素辅助性治疗可减少牙周袋深度和牙周附着丧失程度[[28](https://www-uptodate-cn-s--zhe.cnu100.rbltsg.top/contents/zh-Hans/complications-diagnosis-and-treatment-of-odontogenic-infections/abstract/28)]。有效药物包括2%盐酸米诺环素微球)、10%[盐酸多西环素](https://www-uptodate-cn-s--zhe.cnu100.rbltsg.top/contents/zh-Hans/92300?search=periodontitis&topicRef=3416&source=see_link)缓释液、[氯己定](https://www-uptodate-cn-s--zhe.cnu100.rbltsg.top/contents/zh-Hans/92756?search=periodontitis&topicRef=3416&source=see_link)牙周缓释剂(薄片)和25%甲硝唑凝胶，美国和加拿大无25%甲硝唑凝胶。 |
| 18 | UpToDate:Overview of gingivitis and periodontitis in adults成人牙龈炎和牙周炎概述 | 1、菌斑相关性牙龈炎和牙周炎的治疗—菌斑相关性牙周炎的主要治疗包括由牙科医生进行牙周清洁(针对龈下生物膜和牙石进行刮治和根面平整)，还应进行细致的日常口腔清洁，包括每日使用[氯己定](https://www-uptodate-cn-s--zhe.cnu100.rbltsg.top/contents/zh-Hans/92756?search=periodontitis&topicRef=6858&source=see_link)含漱液2次，待能够安全地恢复正常刷牙后(一般是在刮治或手术后2周)，每日刷牙2次(用手动或电动牙刷)、每日使用牙线1次，并酌情戒烟；  2、吸烟、糖尿病和情绪紧张等因素也会促进牙周炎进展  3、包括一日刷牙2次(手动刷牙或使用电动牙刷)，一日使用牙线1次，以及用抗菌/消毒漱口水含漱 |
| 19 | NICE—1  Type 2 diabetes in adults: management | 1、所有患者口腔健康检查之间的最短间隔应为3个月；  2、牙医应与患者讨论建议的召回间隔，并在当前的记录保存系统中记录此间隔以及患者是否同意。  3、应在下一次口腔健康审查时再次审查召回间隔，以了解患者对所提供的口腔护理的反应和取得的健康结果。该反馈和口腔健康审查的结果应用于调整所选的下一个召回间隔。应告知患者，他们推荐的召回间隔可能会随时间而变化。  对于反复证明他们可以保持口腔健康且不被认为有口腔疾病风险或患口腔疾病的患者，召回间隔可以随着时间的推移延长至24个月。 |
| 20 | NICE-2  Delivering better oral health: an evidence-based toolkit for prevention | 1、快速筛查工具是基础牙周检查 （BPE）[[](https://www.gov.uk/government/publications/delivering-better-oral-health-an-evidence-based-toolkit-for-prevention/chapter-5-periodontal-diseases#fn:50)BPE 使用 WHO BPE 探头，适用于所有齿状成人的常规评估  2、血糖的控制：大多数糖尿病患者的目标 HbA1c 值为 6.5% 或更低；鼓励人们保持良好的糖尿病控制（包括饮食、药物、运动等），并定期与糖尿病医生进行随访  3、风险或易感性和保护因素：  牙菌斑堆积、  烟草：吸烟和使用无烟气烟草制品（询问患者有无吸烟史以及吸烟状况，建议采用最有效的戒烟方法（强推荐）4AA  电子烟;大多数吸电子烟的人以前都是吸烟者，并且会经历吸烟对牙周健康的影响，这是有帮助的  酒精的摄入：减少  未诊断为糖尿病患者的血糖（研究表明患牙周炎患者的糖化比未患牙周炎患者的糖化高）和糖尿病患者的高血糖都是牙周健康状况不佳的危险因素（血糖波动较大）  药物的摄入副作用：口干--抗抑郁的药物；口干牙龈肿大--用于心血管疾病的钙通道阻滞剂  4、采用更详细的牙周图表以识别受影响的部位（患者将分别有 ≥4 毫米或 ≥6 毫米的牙周）牙菌斑评分可以识别需要管理的特定口腔卫生问题的区域  5、每天至少刷牙龈线和每颗牙齿两次（最后一件事是晚上或睡前，以及至少一次其他场合）  6、食物种类的选择：（更健康的饮食习惯）  每天至少吃 5 份各种水果和蔬菜  每天至少吃 5 份各种水果和非淀粉类蔬菜。淀粉类蔬菜（如土豆）只计入碳水化合物摄入量。一份水果或蔬菜是 80 克。新鲜、冷冻、罐装、干燥和榨汁都很重要。一份干果只有 30 克，可以是 3 个杏干或一汤匙葡萄干。重要的是将果汁和冰沙每天的总量限制在 150 毫升。仅一份果汁或冰沙（150 毫升）算作（至少）每天 5 份中的一份。  土豆、面包、米饭、意大利面和其他淀粉类碳水化合物  以淀粉类碳水化合物为基础，包括土豆、面包、米饭和意大利面。选择全麦品种，或将皮留在马铃薯上，以获得更多纤维、维生素和矿物质。  乳制品和替代品  吃一些乳制品或乳制品替代品，但尽可能选择低脂肪的选择。对于酸奶等产品，应鼓励人们查看标签并选择脂肪和糖含量较低的产品。  豆类、豆类、鱼、蛋、肉和其他蛋白质  吃一些豆类、豆类、鱼、蛋、肉和其他蛋白质。每周至少吃 2 份（2 x 140 克）鱼，其中一份是油性的。限制加工肉类，如香肠、培根和腌肉。每天食用超过 90 克红肉或加工肉类的人应尽量将量减少到平均每天不超过 70 克  油和涂抹酱  应鼓励人们谨慎使用这些产品，因为它们的脂肪含量很高。减少这些类型的食物可能有助于控制体重，因为它们的热量很高。  减少高脂肪、高盐和高糖的食物和饮料的摄入量  减少饱和脂肪的最简单方法之一是比较类似产品的标签并选择饱和脂肪含量较低的产品。应鼓励患者注意饱和脂肪含量高的食物，包括脂肪肉、香肠、黄油、奶油、奶酪、巧克力、糕点、蛋糕和饼干。  减少含糖食物和饮料的数量和频率  将含有游离糖的饮料换成水、低脂牛奶或无糖替代品（包括茶和咖啡）特别有帮助  减少盐的摄入量：成人每天应摄入不超过 6 克盐（6 克盐约为一茶匙），儿童应少吃盐。大部分盐已经存在于日常食物中，如面包、早餐麦片、意大利面酱、汤和淀粉类零食  多喝水：建议每天喝大约 6 到 8 杯液体，以防止脱水。水、低脂牛奶和无糖饮料（包括茶和咖啡）都很重要。果汁和冰沙计入液体消耗量，但这是游离糖的来源，因此每天的摄入量应限制在 150 毫升的总量，并建议随餐食用 |
| 21 | PubmedTreatment of stage I-III periodontitis-The EFP S3 level clinical practice guideline | 1、我们建议支持性牙周护理访问应每隔3至最多12个月进行一次，并应根据患者的风险概况和积极治疗后的牙周状况进行调整  2、控制风险因素：戒烟，改善糖尿病的代谢控制，饮食咨询（脂肪摄入、少游离糖和盐摄入、增加水果和蔬菜摄入）  3、我们应该推荐电动牙刷还是手动牙刷？建议（4.5）对于牙周维持患者，可考虑使用电动牙刷替代手动刷牙但是是2018年的  4、如果要辅助使用抗菌牙膏配方，我们建议在牙周炎患者使用含有氯己定、三氯三聚物和氟化亚锡-六偏磷酸钠的产品来控制牙龈炎症  5、如果要辅助使用抗菌漱口水配方，我们建议在牙周炎患者使用含有氯己定、精油和氯化十六烷基吡啶的产品来控制牙龈炎症  6、所有牙周炎患者，不论其疾病阶段，仅适用于失去牙周支持和/或牙周袋形成的牙齿*病因相关治疗）旨在控制（减少/消除）龈下生物膜和牙石（龈下内固定）:  7、使用龈下器械治疗牙周炎，以减少探测袋深度，牙龈炎症和患病部位的数量（证据总结）;干预龈下内固定旨在通过清除牙齿表面的软硬沉积物来减少软组织炎症 |
| 22 | [NICE](https://www.nice.org.uk/guidance/ng30)  [Oral health promotion: general dental practice口腔健康促进指南：一般牙科实践](https://www.nice.org.uk/guidance/ng30)-3  22 | 1、确保建议是量身定制的，以满足个人需求  2、鼓励牙科诊所团队与患者建立良好的关系，以便他们可以帮助患者保持良好的口腔健康。  3、倾听患者的需求并提供量身定制的建议  4、与患者或其父母或护理人员一起制定个性化定制的牙科护理计划 |
| 23 | GIN:Management of Chronic Periodontitis  慢性牙周炎管理 | 1、所有牙周炎患者，不论其疾病阶段，仅适用于失去牙周支持和/或牙周袋形成的牙齿*病因相关治疗）旨在控制（减少/消除）龈下生物膜和牙石（龈下内固定）:  使用龈下器械治疗牙周炎，以减少探测袋深度，牙龈炎症和患病部位的数量（证据总结）;干预龈下内固定旨在通过清除牙齿表面的软硬沉积物来减少软组织炎症 |
| 24 | JBI  Periodontal Disease (Prevention and Treatment): Oral Hygiene Practices | 1、建议使用手动牙刷或电动牙刷。牙刷头小，质地中等。（5级）2023年的证据总结  为了预防和治疗牙周病，应由牙科保健专业人员评估患者及对菌斑或牙间菌斑控制的选择  评估应该包括对消除菌斑及牙间斑治疗方法及辅助器械的选择，以及需要评估患者清除菌斑和目标设定的能力。  2、良好实践是指牙科健康专业人员对患者及其对菌斑控制的偏好进行评估，包括演示刷牙的方法和类型，评估患者清除菌斑的能力，并设定刷牙目标  3、对于有牙周病迹象的患者，建议牙周炎患者每天清洁牙龈 |
| 25 | Periodontitis: Treatment | 1、与牙龈疾病相关的因素：  吸烟；女性激素的改变；糖尿病、癌症及艾滋病、服用抗胆碱能药物（减少唾液流动）、遗传  2、病史：相关的病史，以确定任何潜在的危险因素或者存在的条件（如吸烟）  3、使用手动或电动探针去测量牙周依附状况，这是主要的临床参数，用于评估个人牙周状况 |
| 26 | CINAHL-Consensus report of the joint workshop of the Italian Society of Diabetology, Italian Society of Periodontology and Implantology, Italian Association of Clinical Diabetologists (SID-SIdP-AMD) | 1、注意糖尿病患者的心血管类药物，如阿司匹林、他汀类药物、β受体阻滞剂等对牙科治疗的影响；为糖尿病患者规划预约的时间，麻醉的类型，必要时进行抗生素预防。  2、建议计划和选择最合适的时间进行干预：对糖尿病患者进行拔牙或口腔干预的最佳干预时间为上午中段，早餐后1 ~ 3小时，并可能给予胰岛素剂量。  3、询问患者并检查牙周炎的体征和症状，如牙龈出血、味觉改变、咀嚼疼痛、牙龈肿胀和不适、牙齿活动。 |
| 27 | Pubmed  Scientific evidence on the links between periodontal diseases and diabetes: Consensus report and guidelines of the joint workshop on periodontal diseases and diabetes by the International Diabetes Federation and the European Federation of Periodontology | 1. 症状：牙龈发红或肿胀;刷牙后牙龈出血或水槽中带血;恶臭;牙齿看起来更长;牙齿松动;增加牙齿之间的空间;牙齿上的牙结石（牙垢）。 2. 收集详细的病史，以突出糖尿病的类型、疾病的持续时间、是否存在任何并发症、糖尿病治疗和伴随治疗，请记住，大多数糖尿病患者也在接受抗凝血/抗血小板药物、抗高血压药物或降脂药物治疗。3、糖尿病患者还应评估其他潜在的口腔并发症，包括口干、口灼热、念珠菌感染和龋齿。   4、您需要每天在家中清洁牙齿和牙龈两次，每次至少 2 分钟 |
| 28 | 中华医学知识库  重度牙周炎诊断标准及特殊人群牙周病治疗原则的中国专家共识2017 | 1、糖尿病患者的牙周治疗应根据血糖控制情况及其健康状况实施。  血糖控制理想的患者[空腹血糖4.4～6.1mmol/L，糖化血红蛋白（HbA1c）<6.5%]，牙周治疗同全身健康者。  血糖控制良好的患者（空腹血糖为6.1～7.0mmol/L，HbA1c 6.5%～7.5%），牙周治疗同全身健康者。如需行大范围牙周手术，应合理使用抗生素，术后饮食可咨询内科医师，注意减轻患者的手术焦虑。  血糖控制差（空腹血糖>7.0 mmol/L，HbA1c>7.5%），甚至存在并发症或者使用大剂量胰岛素的患者，建议血糖控制良好后再行牙周治疗。如牙周治疗无法推迟，则仅行牙周基础治疗，可预防性使 用抗生素，慎用含肾上腺素的局麻药，不建议进行牙周手术。  血糖控制极差的患者（空腹血糖>11.4 mmol/L），建议仅做对症急诊处理，待血糖得到有效控制后再行牙周治疗。 2、**治疗时机和时间控制** 牙周治疗推荐安排在上午早饭后和服用降糖药物后约1.5h，治疗时动作应尽量轻柔，治疗时间应控制在2 h以内，避免影响患者的正常饮食。 |
| 29 | Efficacy of different protocols of non-surgical periodontal therapy in patients with type 2 diabetes: A systematic review and meta-analysis | 在糖尿病患者中，NSPT是有效的，并导致牙周临床参数的改善，类似于健康个体的预期，并且与不包括龈下器械的治疗相比，它可以在短期（3-6个月）内改善血糖控制。 |
| 30 | Embase  The effect of antimicrobial photodynamic therapy adjunct to non-surgical periodontal therapy on the treatment of periodontitis in individuals with type 2 diabetes mellitus: A systematic review and meta-analysis | 抗菌光动力治疗配合牙周治疗有助于改善2型糖尿病患者牙周临床参数探诊出血及探诊深度 |
| 31 | Effect of subgingival periodontal therapy on glycaemic control in type 2 diabetes patients: Meta-analysis and meta-regression of 6-month follow-up randomized clinical trials | 牙龈下牙周治疗对T2DM合并牙周炎患者6个月的血糖控制有显著的临床相关改善。 |
| 32 | Efficacy of Antioxidant Supplementation to Non-Surgical Periodontal Therapy on Metabolic Control in Type 2 Diabetes Patients: A Network Meta-Analysis | 与单独使用NSPT相比，补充蜂胶+NSPT是改善 HbA1c 的最有效治疗方法 |
