# Supplementary material for: Summary of the best evidence for non-surgical intervention in periodontitis among patients with type 2 diabetes mellitus
Source: Front Public Health. 2026 Apr 20;14:1792923. doi: 10.3389/fpubh.2026.1792923 (PMC13174932; doi:10.3389/fpubh.2026.1792923)
Supplement: Supplementary file 3 [file Table_3.docx]

| Evidence extracted from the literature | Final evidence | Recommendation level | Grade |
| --- | --- | --- | --- |
| 1. 病史：相关的病史，以确定任何潜在的危险因素或者存在的条件   与牙龈疾病相关的因素：  吸烟；女性激素的改变；糖尿病、癌症及艾滋病、服用抗胆碱能药物（减少唾液流动）、遗传（25）  2、建议收集详细的病史，以突出糖尿病的类型，疾病的持续时间，任何并发症的存在，糖尿病治疗和伴随治疗的到位；应该记住，大多数糖尿病患者也在接受抗凝血/抗聚集剂、降压药和降脂药的治疗。（27） | 病史评估：糖尿病病史评估：糖尿病病程、有无现存的糖尿病并发症，糖尿病治疗方案；以及其他疾病史：癌症及艾滋病；牙周炎遗传史^[25-27]^  Medical History Assessment: Assessment of diabetes history, including the duration of diabetes, the presence of existing diabetic complications, and diabetes treatment regimens; as well as histories of other diseases such as cancer and acquired immunodeficiency syndrome (AIDS); and the family history of periodontitis^[25, 27]^ | 5b | A |
| 1、风险或易感性和保护因素：  牙菌斑堆积、  烟草：吸烟和使用无烟气烟草制品（询问患者有无吸烟史以及吸烟状况，建议采用最有效的戒烟方法  电子烟;大多数吸电子烟的人以前都是吸烟者，并且会经历吸烟对牙周健康的影响，这是有帮助的  酒精的摄入：减少  未诊断为糖尿病患者的血糖（研究表明患牙周炎患者的糖化比未患牙周炎患者的糖化高）和糖尿病患者的高血糖都是牙周健康状况不佳的危险因素（血糖波动较大）  药物的摄入副作用：口干--抗抑郁的药物；口干牙龈肿大--用于心血管疾病的钙通道阻滞剂  2、与牙龈疾病相关的因素：  吸烟；女性激素的改变；糖尿病、癌症及艾滋病、服用抗胆碱能药物（减少唾液流动）、遗传（25）  3、注意糖尿病患者的心血管类药物，如阿司匹林、他汀类药物、β受体阻滞剂等对牙科治疗的影响；为糖尿病患者规划预约的时间，麻醉的类型，必要时进行抗生素预防。（26）  4、收集详细的病史，以突出糖尿病的类型、疾病的持续时间、是否存在任何并发症、糖尿病治疗和伴随治疗，请记住，大多数糖尿病患者也在接受抗凝血/抗血小板药物、抗高血压药物或降脂药物治疗。糖尿病患者还应评估其他潜在的口腔并发症，包括口干、口灼热、念珠菌感染和龋齿。（27） | 危险因素评估：评估有无牙菌斑堆积、吸烟史、饮酒史、服药史（抗抑郁的药物、钙通道阻滞剂、抗凝药物、降压药、他汀类药物、β受体阻滞剂及抗胆碱能药物）^[20,25,26，27]^  Risk Factor Assessment: Evaluate the presence of plaque accumulation, smoking history, alcohol consumption history, and medication history (including antidepressants, calcium channel blockers, anticoagulants, antihypertensive drugs, statins, beta-blockers, and anticholinergic drugs)^[20, 25-27]^ | 4a | **A** |
| 1、推荐所有糖尿病患者每年接受口腔检查。（16）  2、快速筛查工具是基础牙周检查（20） | 建议对所有的糖尿病患者进行基础牙周检查[16,20]  Target Population for Assessment: It is recommended that all patients with diabetes undergo a basic periodontal examination^[16, 20]^. | 5b | A |
| - 1、症状：牙龈发红或肿胀;刷牙后牙龈出血或水槽中带血;恶臭;牙齿看起来更长;牙齿松动;增加牙齿之间的空间;牙齿上的牙结石（牙垢）。 - 2、糖尿病患者还应评估其他潜在的口腔并发症，包括口干、粘膜变化、口灼热、念珠菌感染和龋齿。（27） - 3、询问患者并检查牙周炎的体征和症状，如牙龈出血、味觉改变、咀嚼疼痛、牙龈肿胀和不适、牙齿活动。（26） | 症状评估：检查患者牙龈有无发炎迹象（如肿胀、出血、红肿）、牙齿松动、牙齿敏感、牙齿萎缩或长牙、口臭无法消除、咀嚼疼痛、味觉改变、口干、黏膜改变、龋齿及念珠菌感染的现象^[26，27]^  Examine patients for signs of gingival inflammation (such as swelling, bleeding, and redness), tooth mobility, tooth sensitivity, gingival recession or abnormal tooth eruption, persistent halitosis, chewing pain, taste alterations, xerostomia (dry mouth), mucosal changes, dental caries, and candidal infections^,26，27]^ | 5b | A |
| 1、使用手动或电动探针去测量牙周依附状况，这是主要的临床参数，用于评估个人牙周状况（25）  2、采用更详细的牙周图表以识别受影响的部位（患者将分别有 ≥4 毫米或 ≥6 毫米的牙周）牙菌斑评分可以识别需要管理的特定口腔卫生问题的区域（20） | 评估工具：使用探针判断牙周依附状况；对于已出现牙周炎迹象的患者，应采用更详细的牙周图表和牙菌斑指数以识别需要管理的口腔问题区域^[25,20]；^  Assessment Tools: A probe is used to evaluate the periodontal attachment status; for patients with signs of periodontitis, more detailed periodontal charts and plaque indices should be adopted to identify oral problem areas requiring management^[20, 25]^ | 5b | A |
| 1、良好实践是指牙科健康专业人员对患者及其对菌斑控制的偏好进行评估，包括演示刷牙的方法和类型，评估患者清除菌斑的能力，并设定刷牙目标（24） | 偏好的评估：评估患者对菌斑控制的偏好及清除菌斑能力^[24]^  evaluate the patient's preferences for plaque control and their ability to remove plaque^[24]^ |  | A |
| 1、血糖的控制：大多数糖尿病患者的目标HbA1c值为6.5%或更低（20） | 建议糖尿病患者糖化血红蛋白应≤6.5%  It is recommended that the glycated hemoglobin (HbA1c) level of patients with diabetes should be ≤ 6.5%^[20]^ | 5b | B |
| 1、风险或易感性和保护因素：  烟草：吸烟和使用无烟气烟草制品（询问患者有无吸烟史以及吸烟状况，建议采用最有效的戒烟方法；酒精的摄入：减少（20）  2、控制风险因素：戒烟，改善糖尿病的代谢控制，饮食咨询（脂肪摄入、少游离糖和盐摄入、增加水果和蔬菜摄入）（21）  3、吸烟、糖尿病和情绪紧张等因素也会促进牙周炎进展（18） | 建议患者戒烟、缓解情绪紧张、减少酒精的摄入^[18，20,21]^  It is recommended that patients quit smoking, alleviate emotional stress, and reduce alcohol intake^[18, 20, 21]^ | 1b | A |
| 1、鼓励牙科诊所团队与患者建立良好的关系，以便他们可以帮助患者保持良好的口腔健康。  2、倾听患者的需求并提供量身定制的建议  3、与患者或其父母或护理人员一起制定个性化定制的牙科护理计划（22）  4、良好实践是指牙科健康专业人员对患者及其对菌斑控制的偏好进行评估，包括演示刷牙的方法和类型，评估患者清除菌斑的能力，并设定刷牙目标（24） | 鼓励牙科团队与患者建立良好的关系，了解患者的口腔卫生需求，向患者演示刷牙的方法，根据患者清除菌斑的能力制定个性化牙周护理计划^[22,24]^  Dental teams are encouraged to establish a good relationship with patients, understand their oral hygiene needs, demonstrate toothbrushing techniques to them, and develop personalized periodontal care plans based on the patients' ability to remove dental plaque^[22, 24]^ | 1b | A |
| 1、手动或电动刷牙是减少牙菌斑和牙龈炎的主要方法（21）2018年的证据  2、建议使用手动牙刷或电动牙刷。牙刷头小，质地中等，当刷毛出现脱落、磨损时，及时更换牙刷。（5级）2023年的证据总结（24）  优选23年符合最新发表证据优先的原则进行筛选 | 牙刷的选择：手动或电动牙刷均可，以小刷头，刷毛质地中等为宜，当刷毛出现脱落、磨损时，及时更换牙刷^[21,24]^  Selection of Toothbrushes：  Either manual or electric toothbrushes can be used, with small brush heads and medium-textured bristles being preferred. When the bristles fall out or become worn, the toothbrush should be replaced promptly^[21, 24]^ | 5b | A |
| 1、如果要辅助使用抗菌牙膏配方，我们建议在牙周炎患者支持牙周护理时，使用含有氯己定、三氯三聚物和氟化亚锡-六偏磷酸钠的产品来控制牙龈炎症（21） | 抗菌牙膏的选择：建议在牙周炎患者支持牙周护理时，使用含有氯己定、三氯三聚物和氟化亚锡-六偏磷酸钠的产品来控制牙龈炎症^[21]^  Selection of Antibacterial Toothpaste:  For patients with periodontitis undergoing supportive periodontal care, it is recommended to use products containing chlorhexidine, triclocarban, and stannous fluoride-sodium hexametaphosphate to control gingival inflammation^[21]^ | 1a | **A** |
| 1. 对于有牙周病迹象的患者，建议牙周炎患者每天清洁牙龈（JBI24） 2. 每天至少刷牙龈线和每颗牙齿两次（最后一件事是晚上或睡前，以及至少一次其他场合）（NICE20） 3. 每天在家中清洁牙齿和牙龈两次，每次至少2分钟（27） | 刷牙频次：每天清洁牙齿及牙龈线2次（最后一次清洁是睡前），每次至少2分钟^[20, 24,27]^  Frequency of Toothbrushing:  Teeth and the gingival margin should be cleaned twice a day (with the last cleaning session performed before bedtime)，and each cleaning session should last for at least 2 minutes^[20, 24,27]^ | 5b | A |
| 1、如果解剖学上可能，我们建议在刷牙的同时使用牙间刷  2、我们建议在选择牙刷设计以及选择齿间牙刷设计时，考虑患者的需求和偏好。对于牙刷无法触及的牙间区域，我们建议患者在刷牙时使用其他牙间清洁设备作为补充其他齿间清洁设备包括橡胶/弹性清洁棒、木棒、口腔冲洗器或牙线。  3、当出现牙龈炎症时，应专业教导患者使用牙间刷（idb）进行牙间清洁。（21）  4、建议使用牙间清洁辅助工具，以帮助到达有足够空间的近端表面。牙间刷应紧贴在牙间空间；因此，许多患有牙周炎的人需要大小不同的牙间刷（24）。 | 牙间清洁工具的选择和使用：对于患有牙周炎的患者刷牙前优先选择大小不同的牙间刷清洁牙齿间隙，使用时应紧贴牙间隙；同时也可借助牙线、橡胶、弹性清洁棒、木棒、口腔冲洗器进行齿间清洁^[21，24]^  Selection and Use of Interdental Cleaning Tools:  For patients with periodontitis, before toothbrushing, interdental brushes of different sizes are preferred for cleaning the interdental spaces; when in use, the brushes should be closely pressed against the interdental spaces. Meanwhile, dental floss, rubber or elastic cleaning sticks, wooden sticks, and oral irrigators can also be used for interdental cleaning^[21, 24]^ | 1a | **A** |
| 1、如果要辅助使用抗菌漱口水配方，我们建议在牙周炎患者使用含有氯己定、精油和氯化十六烷基吡啶的产品来控制牙龈炎症（21） | 抗菌漱口水的选择：短期使用含有洗必泰、精油和氯化十六烷基吡啶的产品，以控制牙龈炎症^[21]^  Selection of Antibacterial Mouthwash:  For short-term use, products containing chlorhexidine, essential oils, and cetylpyridinium chloride are recommended to control gingival inflammation^[21]^ | 1a | **A** |
| 1、土豆、面包、米饭、意大利面和其他淀粉类碳水化合物  以淀粉类碳水化合物为基础，包括土豆、面包、米饭和意大利面。选择全麦品种，或将皮留在马铃薯上，以获得更多纤维、维生素和矿物质。(20) | 碳水化合物方面：以淀粉类碳水化合物为主，如带皮土豆、全麦面包、米饭和意大利面，以获得更多纤维、维生素和矿物质^[20]^  Carbohydrate Intake:  Carbohydrate intake should be dominated by starchy carbohydrates, such as potatoes with skin, whole-wheat bread, rice, and pasta, to obtain more fiber, vitamins, and minerals^[20]^ | 5b | B |
| 1、豆类、豆类、鱼、蛋、肉和其他蛋白质  吃一些豆类、豆类、鱼、蛋、肉和其他蛋白质。每周至少吃 2 份（2 x 140 克）鱼，其中一份是油性的。限制加工肉类，如香肠、培根和腌肉。每天食用超过 90 克红肉或加工肉类的人应尽量将量减少到平均每天不超过 70 克  2、乳制品和替代品  吃一些乳制品或乳制品替代品，但尽可能选择低脂肪的选择。对于酸奶等产品，应鼓励人们查看标签并选择脂肪和糖含量较低的产品。(20) | 蛋白质方面：摄入豆类、鱼、蛋、肉及其他蛋白质；每周至少吃280g的鱼，其中一份是油性的；限制加工肉类：香肠、培根和腌肉的摄入；鼓励选择低脂肪的乳制品及替代品^[20]^  Protein Intake:  For protein intake, consume legumes, fish, eggs, meat, and other protein sources. Eat at least 280 grams of fish per week, with one serving being oily fish. Limit the intake of processed meats: sausages, bacon, and cured meats. Encourage the selection of low-fat dairy products and their alternatives^[20]^ | 5b | B |
| 1、油和涂抹酱  应鼓励人们谨慎使用这些产品，因为它们的脂肪含量很高。减少这些类型的食物可能有助于控制体重，因为它们的热量很高。  2、减少高脂肪、高盐和高糖的食物和饮料的摄入量  减少饱和脂肪的最简单方法之一是比较类似产品的标签并选择饱和脂肪含量较低的产品。应鼓励患者注意饱和脂肪含量高的食物，包括脂肪肉、香肠、黄油、奶油、奶酪、巧克力、糕点、蛋糕和饼干。(20) | 脂肪方面：减少油、涂抹酱以及饱和脂肪较高食物的摄入如：奶酪、巧克力、糕点、蛋糕和饼干^[20]^  Fat Intake:  For fat intake, reduce the consumption of oils, spreads, and foods high in saturated fat, such as cheese, chocolate, pastries, cakes, and biscuits^[20]^ | 5b | B |
| 1、每天至少吃 5 份各种水果和非淀粉类蔬菜。淀粉类蔬菜（如土豆）只计入碳水化合物摄入量。一份水果或蔬菜是 80 克。新鲜、冷冻、罐装、干燥和榨汁都很重要。一份干果只有 30 克，可以是 3 个杏干或一汤匙葡萄干。重要的是将果汁和冰沙每天的总量限制在 150 毫升。仅一份果汁或冰沙（150 毫升）算作（至少）每天 5 份中的一份。(20) | 膳食纤维方面：每天至少吃5份各种水果和非淀粉类蔬菜。一份水果约或蔬菜是80克；若水果类型选择干果：干果摄入为30克。若水果以果汁和冰沙的形式存在，每天的总量限制在150毫升^[20]^  Dietary Fiber Intake:  For dietary fiber intake, consume at least 5 servings of a variety of fruits and non-starchy vegetables daily. One serving of fruit or vegetables is approximately 80 grams; if dried fruits are chosen as the fruit type, the intake of dried fruits should be 30 grams. If fruits are consumed in the form of fruit juices or Bsmoothies, the total daily amount should be limited to 150 milliliters^[20]^ | 5b | B |
| 1、多喝水：建议每天喝大约 6 到 8 杯液体，以防止脱水。水、低脂牛奶和无糖饮料（包括茶和咖啡）都很重要。果汁和冰沙计入液体消耗量，但这是游离糖的来源，因此每天的摄入量应限制在 150 毫升的总量，并建议随餐食用,（小组讨论对于糖尿病患者而言不建议果汁随餐食用，建议餐后2小时后食用，因此予以删除）(20)  2、减少含糖食物和饮料的数量和频率  将含有游离糖的饮料换成水、低脂牛奶或无糖替代品（包括茶和咖啡）特别有帮助(20) | 饮料方面：将含糖饮料替换为水、低脂牛奶或无糖替代品（如茶和咖啡），建议每天饮水量为6～8杯^[20]^  Beverage Recommendations:  Replace sugary drinks with water, low-fat milk, or sugar-free alternatives (such as tea and coffee). It is recommended to consume 6 to 8 cups of water daily^[20]^ | 5b | B |
| 1、减少盐的摄入量：成人每天应摄入不超过 6 克盐（6 克盐约为一茶匙），儿童应少吃盐。大部分盐已经存在于日常食物中，如面包、早餐麦片、意大利面酱、汤和淀粉类零食(20) | 盐摄入方面：成人每天应摄入不超过6克盐^[20]^  Salt Intake :  Adults should consume no more than 6 grams of salt per day^[20]^ | 5b | B |
| 1、糖尿病患者的牙周治疗应根据血糖控制情况及其健康状况实施。  血糖控制理想的患者[空腹血糖4.4～6.1 mmol/L，糖化血红蛋白（HbA1c）<6.5%]，牙周治疗同全身健康者。  血糖控制良好的患者（空腹血糖为6.1～7.0 mmol/L，HbA1c 6.5%～7.5%），牙周治疗同全身健康者。如需行大范围牙周手术，应合理使用抗生素，术后饮食可咨询内科医师，注意减轻患者的手术焦虑。  血糖控制差（空腹血糖>7.0 mmol/L，HbA1c>7.5%），甚至存在并发症或者使用大剂量胰岛素的患者，建议血糖控制良好后再行牙周治疗。如牙周治疗无法推迟，则仅行牙周基础治疗，可预防性使 用抗生素，慎用含肾上腺素的局麻药，不建议进行牙周手术。  血糖控制极差的患者（空腹血糖>11.4 mmol/L），建议仅做对症急诊处理，待血糖得到有效控制后再行牙周治疗。(28) | 干预原则：当空腹血糖为4.4～7.0 mmol/L，糖化血红蛋白（HbA1c）<7.5%时，可进行牙周机械干预^[28]^  Principles of Intervention:  When the fasting blood glucose level ranges from 4.4 to 7.0 mmol/L and the glycated hemoglobin (HbA1c) level is less than 7.5%, periodontal mechanical intervention can be performed^[28]^ | 5b | A |
| 1、治疗时机和时间控制 牙周治疗推荐安排在上午早饭后和服用降糖药物后约1.5h，治疗时动作应尽量轻柔，治疗时间应控制在2 h以内，避免影响患者的正常饮食(28)。  2、建议计划和选择最合适的时间进行干预：对糖尿病患者进行拔牙或口腔干预的最佳干预时间为上午中段，早餐后1 ~ 3小时，并可能给予胰岛素剂量（26） | 牙周干预时机：建议最佳机械干预时间为上午中段：早餐后1~3小时、服用降糖药物后约1.5小时。干预时动作应尽量轻柔，时间应控制在2小时以内^[26,28]^  Timing of Periodontal Intervention:  The optimal time for mechanical intervention is recommended to be mid-morning: 1 to 3 hours after breakfast and approximately 1.5 hours after taking hypoglycemic medications. During the intervention, movements should be as gentle as possible, and the duration should be controlled within 2 hours^[26, 28]^ | 5b | A |
| 1、所有牙周炎患者，不论其疾病阶段，仅适用于失去牙周支持和/或牙周袋形成的牙齿病因相关治疗）旨在控制（减少/消除）龈下生物膜和牙石（龈下内固定）:  2、使用龈下器械治疗牙周炎，以减少探测袋深度，牙龈炎症和患病部位的数量（证据总结）;干预龈下内固定旨在通过清除牙齿表面的软硬沉积物来减少软组织炎症（21）  3、所有牙周炎患者，不论其疾病阶段，仅适用于失去牙周支持和/或牙周袋形成的牙齿病因相关治疗）旨在控制（减少/消除）龈下生物膜和牙石（龈下内固定）:  4、使用龈下器械治疗牙周炎，以减少探测袋深度，牙龈炎症和患病部位的数量（证据总结）;干预龈下内固定旨在通过清除牙齿表面的软硬沉积物来减少软组织炎症（23）  在糖尿病患者中，NSPT 是有效的，并导致牙周临床参数的改善，类似于健康个体的预期，并且与不包括龈下器械的治疗相比，它可以在短期（3-6 个月）内改善血糖控制。（29）  牙龈下牙周治疗对T2DM合并牙周炎患者6个月的血糖控制有显著的临床相关改善。（31） | 建议在24h内行机械干预（龈下刮治和根面平整术）以去除牙结石、牙菌斑和病变牙骨质，有效控制牙周炎症，改善血糖水平^[21,23,30,32]^  It is recommended to perform mechanical intervention (subgingival scaling and root planing) within 24 hours to remove dental calculus, dental plaque, and diseased cementum, thereby effectively controlling periodontal inflammation and improving blood glucose levels^[21, 23, 29, 31]^ | 1a | **High** |
| 1、菌斑相关性牙龈炎和牙周炎的治疗 — 菌斑相关性牙周炎的主要治疗包括由牙科医生进行牙周清洁(针对龈下生物膜和牙石进行刮治和根面平整)，还应进行细致的日常口腔清洁，包括每日使用[氯己定](https://www-uptodate-cn-s--zhe.cnu100.rbltsg.top/contents/zh-Hans/92756?search=periodontitis&topicRef=6858&source=see_link)含漱液2次，待能够安全地恢复正常刷牙后(一般是在刮治或手术后2周)，每日刷牙2次(用手动或电动牙刷)、每日使用牙线1次，并酌情戒烟（18） | 机械性干预后，应每日使用[氯己定](https://www-uptodate-cn-s--zhe.cnu100.rbltsg.top/contents/zh-Hans/92756?search=periodontitis&topicRef=6858&source=see_link)含漱液2次，待恢复正常刷牙后(一般是在刮治后2周)，每日刷牙2次(用手动或电动牙刷)、每日使用牙线1次，并酌情戒烟^[18]^  After mechanical intervention, chlorhexidine mouthwash should be used twice a day. Once normal toothbrushing is resumed (typically 2 weeks after scaling), brush teeth twice a day (using a manual or electric toothbrush), use dental floss once a day, and quit smoking as appropriate^[18]^ | 1a | **A** |
| 1、在牙科诊所准备一个“急救包”，包括：测量血糖的屈光计、超快速胰岛素、胰高血糖原、Hypokit 糖包卡波顿。（26） | 急救物品的配备：牙科诊疗室内应配有急救包，急救包内备有血糖仪、超速效胰岛素、胰高血糖素注射剂、糖块等^[26]^  A first-aid kit should be available in the dental clinic, and the kit should contain a blood glucose meter, ultra-rapid-acting insulin, glucagon injection, sugar cubes, and other items^[26]^ | 5b | A |
| 1、抗菌光动力治疗配合牙周治疗有助于改善2型糖尿病患者牙周临床参数探诊出血及探诊深度（30） | 在机械干预时可采用抗菌光动力疗法以改善患者牙周状况^[30]^  During mechanical intervention, antibacterial photodynamic therapy (aPDT) can be applied to improve the patient's periodontal condition^[30]^ | 1a | **B** |
| 1、与单独使用 NSPT 相比，补充蜂胶+NSPT 是改善 HbA1c 的最有效治疗方法（32） | 在机械干预期间，建议适当补充蜂胶制剂，可有效改善血糖水平^[32]^  During mechanical intervention, it is recommended to appropriately supplement propolis preparations, which can effectively improve blood glucose levels^[32]^ | 1a | **B** |
| **1、成人非重度牙周炎**−对于非重度成人牙周炎(2017年牙周病和种植体周围病国际分类根据病情的严重程度及管理复杂性归为Ⅰ期或Ⅱ期，可在刮治+根面平整的同时，在龈下放置局部用抗生素制剂、释放一定量的抗生素，以减少牙周袋深度。据报道，这种局部抗生素辅助性治疗可减少牙周袋深度和牙周附着丧失程度。有效药物包括2%盐酸米诺环素微球)、10%[盐酸多西环素](https://www-uptodate-cn-s--zhe.cnu100.rbltsg.top/contents/zh-Hans/92300?search=periodontitis&topicRef=3416&source=see_link)缓释液、[氯己定](https://www-uptodate-cn-s--zhe.cnu100.rbltsg.top/contents/zh-Hans/92756?search=periodontitis&topicRef=3416&source=see_link)牙周缓释剂(薄片)和25%甲硝唑凝胶，美国和加拿大无25%甲硝唑凝胶。（17） | 在机械干预时，建议在龈下放置局部用抗生素制剂如2%盐酸米诺环素微球、10%[盐酸多西环素](https://www-uptodate-cn-s--zhe.cnu100.rbltsg.top/contents/zh-Hans/92300?search=periodontitis&topicRef=3416&source=see_link)缓释液、[氯己定](https://www-uptodate-cn-s--zhe.cnu100.rbltsg.top/contents/zh-Hans/92756?search=periodontitis&topicRef=3416&source=see_link)牙周缓释剂和25%甲硝唑凝胶，以减少牙周袋深度和牙周附着丧失程度^[17]^  During mechanical intervention, it is recommended to place local antibiotic preparations subgingivally, such as 2% minocycline hydrochloride microspheres, 10% doxycycline hydrochloride sustained-release solution, chlorhexidine periodontal sustained-release agents, and 25% metronidazole gel, to reduce the depth of periodontal pockets and the degree of periodontal attachment loss^[17]^ | 1a | **B** |
| 1、我们建议支持性牙周护理的就诊应安排在3个月至最长12个月之间，并应根据患者的风险状况和积极治疗后的牙周状况进行调整（21）  2、所有患者口腔健康检查之间的最短间隔应为3个月；  牙医应与患者讨论建议的召回间隔，并在当前的记录保存系统中记录此间隔以及患者是否同意。  3、应在下一次口腔健康审查时再次审查召回间隔，以了解患者对所提供的口腔护理的反应和取得的健康结果。该反馈和口腔健康审查的结果应用于调整所选的下一个召回间隔。应告知患者，他们推荐的召回间隔可能会随时间而变化。  对于反复证明他们可以保持口腔健康且不被认为有口腔疾病风险或患口腔疾病的患者，召回间隔可以随着时间的推移延长至24个月（19）  基于整合建议，因为糖尿病患者随着血糖水平的变化牙周炎的严重程度也随之变化，糖尿病患者对于血糖的控制，三个月复查糖化血红蛋白，对于控制较好的患者加你6个月，甚至是一年为最宽时限，因此3-12个月较为保险 | 访视间隔：根据牙周治疗结果和牙齿疾病风险的评估，为每位患者确定口腔健康检查间隔时间，就诊访视间隔时间为3-12个月^[19,21]^  Interval Between Visits:  Based on the outcomes of periodontal treatment and the assessment of dental disease risk, the interval for oral health check-ups shall be determined for each patient, with the interval between clinical visits ranging from 3 to 12 months^[19, 21]^ | 5b | A |
